# Modeling Psychometric Relational Data in Social Networks: Latent Interdependence Models

**DOI:** 10.3389/fpsyg.2022.860837

**Published:** 2022-04-07

**Authors:** Bo Hu, Jonathan Templin, Lesa Hoffman

**Affiliations:** ^1^Department of Applied Psychology, Ningbo University, Ningbo, China; ^2^Department of Psychological and Quantitative Foundations, University of Iowa, Iowa City, IA, United States

**Keywords:** psychometric models, relationship measurement, social networks, latent inter-dependence models, Bayesian estimation

## Abstract

In the current paper, we propose a latent interdependence approach to modeling psychometric data in social networks. The idea of latent interdependence is adopted from social relations models (SRMs), which formulate a mutual-rating process by both dyad members’ characteristics. Under the framework of the latent interdependence approach, we introduce two psychometric models: The first model includes the main effects of both rating-sender and rating-receiver, and the second model includes a latent distance effect to assess the influence from the dissimilarity between the latent characteristics of both sides. The latent distance effect is quantified by the Euclidean distance between both sides’ trait scores. Both models use Bayesian estimation *via* Markov chain Monte Carlo. How accurately model parameters were estimated was evaluated in a simulation study. Parameter recovery results showed that all parameters were accurately recovered under most of the conditions investigated. As expected, the accuracy of model estimation was significantly improved as network size grew. Also, through analyzing empirical data, we showed how to use the estimates of model parameters to predict the latent weight of connections among group members and rebuild either a univariate or multivariate network at a latent trait level. Finally, we discuss issues regarding model comparison and offer suggestions for future studies.

## Introduction

Social and behavioral scientists use the word *relation* to portray the way in which individuals or other social entities (e.g., organizations or countries) are connected. Conceptually, a relation reveals the relevance of one entity to another, and its fundamental unit is a *dyadic interaction*. Relations are sometimes simply defined as connections or links between dyad members, and at other times by various relational constructs (e.g., collaboration and attachment). In the latter condition, researchers assess these constructs by observing dyad members’ responses to a set of well-designed items, inferring each member’s construct scores *via* observed psychometric relational data. Within these measurement settings, relations are practically treated as individuals’ personal traits, defined as a person’s attitude toward or willingness and actions to develop a certain type of interpersonal interaction with others.

Modeling and analyzing relational data are the goals of two major statistical analysis paradigms: dyadic data analysis (DDA) and social network analysis (SNA). The focus of DDA (e.g., the social relations model, Kenny and La Voie, 1984; Kashy and Kenny, 1990; Nestler, 2018; Nestler et al., 2020) is on quantifying *non-independence* through a partitioning of the variance observed in dyadic data (Kenny et al., 2006). SNA, in contrast, attempts to explain the distributions of observed ties (or weighted ties) among social entities using a series of models (e.g., the p* models, the exponential random graphs models, and the latent space models, Wasserman and Pattison, 1996; Pattison and Wasserman, 1999; Hoff et al., 2002; Snijders et al., 2006; Sewell and Chen, 2015). Despite having been considered as routine methodological choices by social and behavioral researchers in studying relations, both approaches are limited in modeling psychometric data representing relational constructs (e.g., interpersonal trust) with potentially complex latent structures due to the insufficiency of psychometric models within their formulations (Snijders, 2009).

In recent years, the efforts to combine network analysis and psychometrics could be seen in attempts to apply the idea of network modeling in psychometrics (e.g., Schmittmann et al., 2013; Epskamp et al., 2017; Jin and Jeon, 2019; Jeon et al., 2021). For instance, Epskamp et al. (2017) introduced a framework to incorporate network modeling into structural equation modeling. The framework features latent network modeling and residual network modeling, in which the covariance structures of both latent variables and residuals are explained as the results of the interplay of each pair of latent or residual components. These efforts advanced the development and application of psychological networks (e.g., Cramer et al., 2010), in which nodes are variables. Also, in analyzing item response data, Jin and Jeon (2019) proposed a joint network modeling approach to detect the local dependence among items and among test-takers. By adapting a latent space approach (Hoff et al., 2002), they constructed multi-layer item networks and multi-layer person networks from item response data, which are modeled as a function of the latent positions of items or persons. Although researchers combined network and psychometric modeling, these approaches did not focus on modeling psychometric relational data within networks, as in the present study.

Recent work by Nestler and colleagues (e.g., Nestler, 2018; Nestler et al., 2020) under the framework of the social relations model (SRM) have made significant advances in modeling psychometric relational data. Specifically, they extended the classic SRM from single-item to multivariate settings and proposed social relations structural equation models (SR-SEM) for data coming from “multiple” round-robin designs (i.e., that require mutual ratings on multiple items). In SR-SEM, person-level SRM effects (or true SRM effects) function as latent factors in explaining item-level SRM effects. They represent a person’s general tendency to perceive others in a certain way (the true actor/perceiver effect), their general tendency to be perceived by others in a certain way (the true partner/target effect), and the true uniqueness of a given dyad’s mutual ratings. From a psychometric standpoint, SR-SEM produces estimates for each person’s two traits (i.e., their general tendencies to perceive others and to be perceived by others in a certain way) and for each dyad’s uniqueness. However, it does not directly provide estimates for the relational construct that the items are meant to measure. As aforementioned, when defined as a personal trait, a relational construct describes one’s attitude toward or willingness and actions to develop or maintain a certain type of interpersonal interaction with others. For instance, consider a scenario in which a researcher created a few items (e.g., “*I can rely on my partner.*”) to measure a relational construct *interpersonal trust* and administered them to a group of individuals. Using the SR-SEM, the researcher could estimate everyone’s general tendency to be trusted and that to trust others, and every dyad’s relational uniqueness. However, it should be noted that these two tendencies (i.e., one’s trustfulness and trustworthiness) from SR-SEM are not the relational construct of *interpersonal trust* itself, which, as a personal trait, is defined as one’s attitude to engaging in a mutual trust with others. Those who score higher on this trait feel more positive about mutual trust and show a stronger tendency to develop or maintain a mutual trust with others.

Moreover, in modeling dyadic data, traditional approaches (e.g., SRM and SNA) give less attention to the characteristics of an item. However, in a round-robin design, for example, dyadic data may be generated from both person-focused items (e.g., *“I like my partner.”*) and dyad-focused items (e.g., *“My partner and I [or We] like each other”*). In fact, it is common to see both types of items from the same instrument [e.g., McAllister’s (1995) Interpersonal Trust Measure; Horvath and Greenberg’s (1989) Working Alliance Inventory]. For interpersonal relationship researchers, understanding what these differences mean in terms of measuring a relational construct is of practical importance for the relationship assessment.

The goal of this paper is to present and evaluate a set of new psychometric models—latent interdependence models (LIDM)—that describe continuous dyadic relational data within a social network. The models aim to score each person’ traits directly along each latent dimension of a relational construct while evaluating the properties of the items that measure each trait. We introduce two models under the framework of LIDM, in which the first model is nested within the second one. We establish the link between group members’ responses to an item and their latent relational traits based on an understanding that the responses come from a mutual-rating process and therefore reflect the characteristics of both members in a dyad. To formulate this fundamental process, in Model 1, we explain the dyadic responses using the main effects of both dyad members’ latent traits. Model 2 then builds on Model 1 to formulate the influence from the dissimilarity between dyad members as a penalty by including a testable model component for the distance between dyad members’ latent traits.

The influence of dyad members’ characteristics and the dissimilarity between dyad members on their responses to a given item is also conditional on the item’s properties—namely, its sensitivity to a change of trait scores and to a change in the distance between dyad members’ scores. In what follows, we define these properties as the item-specific rating-sender effect (i.e., the effect of rating-sender’s latent traits on all responses to a given item), the item-specific rating-receiver effect (i.e., the effect of rating-receiver’s trait on all responses to a given item), and the item-specific latent distance effect (i.e., the effect of the dissimilarity between latent traits on all responses to a given item).

The view that dyadic data come from a mutual-rating process and are therefore interdependent was initially formulated in the social relation model (SRM, e.g., Kenny and La Voie, 1984; Kashy and Kenny, 1990), which has been used to analyze social network data from a round-robin design (Back and Kenny, 2010). The SRM takes an approach similar to that of analysis of variance, assuming observed variances come from three sources (a.k.a. “three SRM effects”): the rating sender’s general rating tendency (the actor effect), the rating receiver’s general rating-reception tendency (the partner effect), and the uniqueness of a given sender-receiver dyad (the relational effect). In the LIDM proposed in the present study, the notion of interdependence is formulated by including the latent traits of both the rating sender and the rating receiver as explanatory components for dyad members’ responses to an item.

Another presumed mechanism underlying mutual-rating processes was described by Hoff et al. (2002) based on the view that the closer two group members’ latent positions are in a social space, the more likely it is that a tie exists between them. Accordingly, they presented latent space models (LSM), where *social space* is a hypothetical concept, defined as “a space of unobserved latent characteristics that represent potential transitive tendencies in network relations” (p. 1091), and latent positions are abstract points in the hypothetical space that indicate group members’ relative positions. Under the framework of LIDM, we assume the rule suggested by LSM also function under the mutual-rating process, by which we build Model 2 (with between-trait interaction) on Model 1 (with main effects of traits only). In Model 2, we assume the relative spatial positions of rating senders and rating receivers along latent dimensions account for a unique proportion of variance of both sides’ responses; we term their contributions as *latent distance effects*. As such, Model 1—with latent distance effects constrained to be zero—is nested within Model 2. The latent distance is operationally defined as the dissimilarity between two members’ latent traits.

The remainder of this paper is organized as follows. We first introduce a scenario in which the proposed LIDM would apply. We then describe the development of the two proposed models in more detail, as well as the Bayesian estimation using Markov chain Monte Carlo (MCMC) algorithm with which to estimate their parameters. We then present a simulation study to evaluate how accurately each proposed model can be estimated. We then show *via* an illustrative example of real data how to use each model variant. We conclude with recommendations for model selection and future research on LIDM development.

## The Latent Interdependence Models

To illustrate the scenario to which the LIDM are intended to apply, we present a portion of the interpersonal trust survey data in Table 1, which were collected as part of a course evaluation protocol. Respondents were 15 graduate students (11 females and 4 males) from a master’s program in Applied Psychology who attended a group therapy course at a comprehensive university in central China. The trust survey was adapted from McAllister’s (1995) Interpersonal Trust Measure (ITM). Three items were selected from the ITM’s affect-based trust (ABT) subscale and were administered to students at the second to last meeting. A sample ABT item is “We have a sharing relationship. We can both freely share our ideas, feelings, and hopes.” The other three items were selected from its cognition-based trust (CBT) subscale and were completed by students at the last meeting. A sample CBT item is “I can rely on this person not to make my job more difficult by careless work.” Students rated their agreement with each statement on a 7-point Likert scale ranging from 1 (Strongly disagree) to 7 (Strongly agree) about each individual classmate in the class. Students’ responses were treated as continuous variables. In Table 1, the first two columns describe the structure of a network, and the remainder of the columns holds the psychometric responses indicating the two latent dimensions of interpersonal trust. In the following sections, we first introduce the format of a network and the format of psychometric data in a network.

**TABLE 1:** An illustrative portion of the interpersonal trust survey data.

Dyads	Direction	ABT_1	ABT_2	ABT_3	CBT_1	CBT_2	CBT_3
(1,2)	1→2	3	4	2	4	2	4
	2→1	5	3	4	3	3	5
(1,3)	1→3	3	3	4	4	3	3
	3→1	5	4	3	1	2	2
(1,4)	1→4	3	4	3	2	3	4
	4→1	2	3	2	3	5	1
(2,3)	1→5	3	4	2	4	3	3
	5→1	3	5	4	4	5	4
(2,4)	2→4	6	3	4	1	3	3
	4→2	3	4	3	3	4	3
(3,4)	3→4	4	4	6	3	4	5
	4→3	3	4	3	3	4	4

*ABT, affect-based trust; CBT, cognition-based trust.*

### Social Networks

For a social network that consists of *n* members, the number of dyads in the network is $\left(\begin{array}{c} n\\ 2 \end{array}\right)$. Let *y*_*A,B*_ denote the relations between group members, *A*, *B* = 1,…, *n*, *A* ≠ *B*. Conventionally, the social network data are represented by an *n* × *n* sociomatrix ***Y***,


$$\text{Y}=\left[\begin{array}{cc} \begin{array}{cc} 0 & y_{1,2}\\ y_{2,1} & 0 \end{array} & \begin{array}{cc} y_{1,3} & \begin{array}{cc} & y_{1,n} \end{array}\\ y_{2,3} & \begin{array}{cc} & y_{2,n} \end{array} \end{array}\\ \begin{array}{cc} \begin{array}{c} y_{3,1}\\ \vdots \\ y_{n,1} \end{array} & \begin{array}{c} y_{3,2}\\ \vdots \\ y_{n,2} \end{array} \end{array} & \begin{array}{cc} 0 & \begin{array}{cc} & y_{3,n} \end{array}\\ \begin{array}{c} \vdots \\ y_{n,3} \end{array} & \begin{array}{c} \begin{array}{cc} \ddots & \vdots \end{array}\\ \begin{array}{cc} \ldots & 0 \end{array} \end{array} \end{array} \end{array}\right],$$

where *y*_*A,B*_ can be any type of observed variable, and *y*_*A,B*_ = 0 by convention for *A* = *B*. The social network data can also be thought of as a graph in which the nodes are group members and the edge set is {(*A*,*B*):*y*_*A*,*B*_≠0}. Of relevance for the present study, *y*_*A,B*_ can also be latent variables representing the magnitude of member’ perceived connections with others along a given latent dimension of a relational construct. In this present case, *y*_*A,B*_ needs to be inferred through observed psychometric relational data in social networks, as described next.

### Psychometric Relational Data in Social Networks

Let *d* = 1,2,…,$\left(\begin{array}{c} n\\ 2 \end{array}\right)$ be the *d*th dyad of the network, *l* = 1,2,…, *L* be the *l*th subscale, and *i* = 1, 2,…, *I* be the *i*th item in the subscale. Then, the responses from the *l*th subscale for the *d*th dyad of members *A* and *B*, are contained in an *I* × 2 dyadic matrix ***U***. The first column of ***U*** contains the responses of *A*, and the second column contains the responses of *B*. Considering a situation in which all subscales have an equal number of items, the dyadic responses of both members to all items can be expressed as a *T* × 2 matrix, **V**, *T* = *I* × *L*.


$$\text{U}=\left[\begin{array}{cc} y_{1\,A\,B}^{l} & y_{1\,B\,A}^{l}\\ \begin{array}{c} y_{2\,A\,B}^{l}\\ y_{3\,A\,B}^{l}\\ \begin{array}{c} \vdots \\ y_{I\,A\,B}^{l} \end{array} \end{array} & \begin{array}{c} y_{2\,B\,A}^{l}\\ y_{3\,B\,A}^{l}\\ \begin{array}{c} \vdots \\ y_{I\,B\,A}^{l} \end{array} \end{array} \end{array}\right]\;\text{V}=\left[\begin{array}{cc} y_{1\,A\,B}^{1} & y_{1\,B\,A}^{1}\\ \begin{array}{c} y_{2\,A\,B}^{1}\\ y_{3\,A\,B}^{1}\\ \begin{array}{c} \vdots \\ \begin{array}{c} y_{I\,A\,B}^{1}\\ \vdots \\ \begin{array}{c} \vdots \\ y_{1\,A\,B}^{L}\\ \begin{array}{c} y_{2\,A\,B}^{L}\\ y_{3\,A\,B}^{L}\\ \begin{array}{c} \vdots \\ y_{I\,A\,B}^{L} \end{array} \end{array} \end{array} \end{array} \end{array} \end{array} & \begin{array}{c} y_{2\,B\,A}^{1}\\ y_{3\,B\,A}^{1}\\ \begin{array}{c} \vdots \\ \begin{array}{c} y_{I\,B\,A}^{1}\\ \vdots \\ \begin{array}{c} \vdots \\ y_{1\,B\,A}^{L}\\ \begin{array}{c} y_{2\,B\,A}^{L}\\ y_{3\,B\,A}^{L}\\ \begin{array}{c} \vdots \\ y_{I\,B\,A}^{L} \end{array} \end{array} \end{array} \end{array} \end{array} \end{array} \end{array}\right],$$

where $y_{iAB}^{l}$ and $y_{iBA}^{l}$ are member *A*’s responses relating to member *B* and *B*’s responses relating to *A*, respectively, to the *i*th item that measures the *l*th latent dimension. Let **v1** and **v2** be the two column vectors of **V**. We assume both vectors follow the same multivariate Gaussian density:


$$\mathrm{v1}\,\text{and}\,\mathrm{v2}∼N\,\left(\mu_{y},\Sigma_{T}\right),$$

where **Σ**_T_ is a *T × T* variance–covariance matrix.

### Model Development

To present the latent interdependence models (LIDM), we first describe two general models to formulate the interdependence process within a network. We then introduce two latent interdependence models (named as Model 1 and Model 2) as the constrained form of these two general models. Both the general models and their constrained forms (i.e., LIDM) assume that group members’ responses are conditionally independent given their latent trait scores, the dyads they are embedded in, and the items they respond to. In building the general models and LIDM, we consider situations in which each item only measures one latent trait, and we denote $\theta_{A}^{l}$ and $\theta_{B}^{l}$ as group member *A*’s and *B*’s latent trait scores on the *l*th latent dimension, respectively. For instance, in the interpersonal trust example, θ_*A*_ and θ_*B*_ could represent any pair of students’ trait scores on either ABT or CBT dimension.

To formulate the interdependence process, in the first general model, two dyad members’ observed responses are predicted using a linear function, in which a member’s response to an item depends on both members’ scores on the latent trait measured by the item, the shared effect of two members from the dyad they both belong to, as well as the item effect due to all group members repeatedly responding to and being rated on the same item in a round-robin setting. That is,


(1) $$y_{i\,A\,B}^{l}=\beta_{0}+\beta_{1\,i\,A\,B}^{l}\,\theta_{A}^{l}+\beta_{2\,i\,A\,B}^{l}\,\theta_{B}^{l}+\alpha_{i\,A\,B}^{l}+\pi_{i}^{l}+\varepsilon_{i\,A\,B}^{l}$$

(2) $$y_{i\,B\,A}^{l}=\beta_{0}+\beta_{1\,i\,B\,A}^{l}\,\theta_{B}^{l}+\beta_{2\,i\,B\,A}^{l}\,\theta_{A}^{l}+\alpha_{i\,A\,B}^{l}+\pi_{i}^{l}+\varepsilon_{i\,B\,A}^{l},$$

where β_0_ is the grand mean of all responses by all group members, $\beta_{1\,i\,A\,B}^{l}$ ($\beta_{1\,i\,B\,A}^{l}$) and $\beta_{2\,i\,A\,B}^{l}\,\left(\beta_{2\,i\,B\,A}^{l}\right)$ are the dyad-specific effects of one dyad member’s and the other’s latent trait, respectively, $\alpha_{i\,A\,B}^{l}$ is the dyad-specific effect shared by both members on the given item, $\pi_{i}^{l}$ is the item effect encapsulating two components for the effect from the *i*th item on all rating senders and that on all rating receivers, respectively, and $\varepsilon_{i\,A\,B}^{l}$ and $\varepsilon_{i\,B\,A}^{l}$ are the residuals for each pair of ratings.

The model is presented in a dyadic format, in which Function (1) explains dyad member A’s observed rating $y_{iAB}^{l}$ to member B, and Function (2) explains the process that generates B’s observed rating $y_{iBA}^{l}$ to A. Note that a member’s latent trait, $\theta_{A}^{l}$ for instance, has different effects on the observed responses in Function (1) and (2), for in a mutual-rating process a member needs to play two different roles—as both a rating sender and a rating receiver. For example, in Function (1), where member A plays the rating sender, $\theta_{A}^{l}$ contributes to A’s observed response through a *rating-sender effect*. In contrast, in Function (2), where A plays a rating receiver, $\theta_{A}^{l}$ contributes to B’s response through a *rating-receiver effect*. For this reason, to estimate $\theta_{A}^{l}$, observed responses from both A and B are needed.

The rationale behind this formulation is that a relation reveals relevance of one to another, and therefore items designed to measure a relational construct in any dyadic (e.g., a round robin design) or network settings should be reflective of a dyadic combination, rather than the characteristics of an individual alone. In the interpersonal trust example, students’ responses to the item “We have a sharing relationship” [extracted from McAllister’s (1995) *Interpersonal Trust Measure*] designed to measure affect-based trust, should be reflective of both one’s own trait as the rating sender and that of the rating receiver.

The second general model formulates the interdependence process to include the dissimilarity between dyad members’ characteristics as a penalty, such that a dyad member’s response to an item is a function of both members’ latent trait scores, the Euclidean distance between their latent trait scores, and the item effect:


(3) $$y_{i\,A\,B}^{l}=\beta_{0}+\beta_{1\,i\,A\,B}^{l}\,\theta_{A}^{l}+\beta_{2\,i\,A\,B}^{l}\,\theta_{B}^{l}+\beta_{3\,i\,A\,B}^{l}\,E_{d}+\pi_{i}^{l}+\varepsilon_{i\,A\,B}^{l}$$

(4) $$y_{i\,B\,A}^{l}=\beta_{0}+\beta_{1\,i\,B\,A}^{l}\,\theta_{B}^{l}+\beta_{2\,i\,B\,A}^{l}\,\theta_{A}^{l}+\beta_{3\,i\,B\,A}^{l}\,E_{d}+\pi_{i}^{l}+\varepsilon_{i\,B\,A}^{l}$$

where *E_d_* is the Euclidean distance between two members of the *d*th dyad and $\beta_{3\,i\,A\,B}^{l}$ ($\beta_{3\,i\,B\,A}^{l}$) is the dyad specific effect of the distance metric related to different members. For $\theta_{A}^{l}$ and $\theta_{B}^{l}$∈ℝ^*N*^,


(5) $$E_{d}={\left(\sum_{l=1}^{L}{\left(\theta_{A}^{l}-\theta_{B}^{l}\right)}^{2}\right)}^{1/2},$$

and *E*_*d*_=|θ_*A*_-θ_*B*_| for *L* = 1. A correlation between sender effects (e.g., $\beta_{1\,i\,A\,B}^{l}$) and receiver effects (e.g.,$\beta_{2\,i\,B\,A}^{l}$) associated with the same trait is allowed across dyads, suggesting that the role a rating sender’s trait plays in their ratings to receivers may correlate with the role the same trait plays in the receivers’ ratings to the sender.

The latent interdependence models (LIDM) are constrained versions of more general models. To describe these constraints, we first define $\beta_{1\,i\,A\,B}^{l}$ and $\beta_{1\,i\,B\,A}^{l}$ as sender effects and $\beta_{2\,i\,A\,B}^{l}$ and $\beta_{2\,i\,B\,A}^{l}$ as receiver effects. We then constrain these receiver effects to be positive and the variance of the receiver effects to be one to identify the covariance matrix for the sender- and receiver- effects. Also, we constrain the value of $\beta_{3\,i\,A\,B}^{l}$ to be negative with an assumption that the distance metric always has a negative contribution to the response, as also stated in Hoff et al.’s (2002, p. 1091) latent space models. Further, we assume that, within a dyad, $\beta_{1\,i\,A\,B}^{l}$ = $\beta_{1\,i\,B\,A}^{l}$, $\beta_{2\,i\,A\,B}^{l}$ = $\beta_{2\,i\,B\,A}^{l}$, $\beta_{3\,i\,A\,B}^{l}$ = $\beta_{3\,i\,B\,A}^{l}$, and $\varepsilon_{i\,A\,B}^{l}$ = $\varepsilon_{i\,B\,A}^{l}$. Finally, sender effects, receiver effects, distance metric effects, and the variance of the residuals are assumed to be invariant across dyads and only to vary across items. Following these imposed restrictions, a correlation between item-specific sender effects and item-specific receiver effects associated with the same trait is allowed across items for LIDM.

As Model 1, the first constrained form of the LIDM can be written as:


(6) $$y_{i\,A\,B}^{l}=\beta_{0}+\beta_{1\,i\,A\,B}^{l}\,\theta_{A}^{l}+\beta_{2\,i\,A\,B}^{l}\,\theta_{B}^{l}+\xi_{i}^{l}$$

(7) $$y_{i\,B\,A}^{l}=\beta_{0}+\beta_{1\,i\,B\,A}^{l}\,\theta_{B}^{l}+\beta_{2\,i\,B\,A}^{l}\,\theta_{A}^{l}+\xi_{i}^{l}$$

where $\xi_{i}^{l}$ = $\alpha_{i\,A\,B}^{l}$ + $\pi_{i}^{l}$$\varepsilon_{i}^{l}$ is the item-specific residual term. We present Model 2 as the second constrained form of the LIDM, which can be written as:


(8) $$y_{i\,A\,B}^{l}=\beta_{0}+\beta_{1\,i\,A\,B}^{l}\,\theta_{A}^{l}+\beta_{2\,i\,A\,B}^{l}\,\theta_{B}^{l}+\beta_{D\,i}^{l}\,E_{d}+\xi_{i}^{l}$$

(9) $$y_{i\,B\,A}^{l}=\beta_{0}+\beta_{1\,i\,B\,A}^{l}\,\theta_{B}^{l}+\beta_{2\,i\,B\,A}^{l}\,\theta_{A}^{l}+\beta_{D\,i}^{l}\,E_{d}+\xi_{i}^{l}$$

where $\xi_{i}^{l}$ = $\pi_{i}^{l}$ + $\varepsilon_{i}^{l}$, and $\beta_{D\,i}^{l}$ is the item-specific effect of the distance metric.

Let *S* represent the person providing the rating (the Sender) and *R* represent the person being rated (the Receiver). We define $y_{iSR}^{l}$ as an observed response of any rating sender to an item from the *l*th subscale for any rating receiver. Accordingly, $\theta_{S}^{l}$ and $\theta_{R}^{l}$, respectively, represent a rating sender’s and a rating receiver’s trait, and $\beta_{S\,i}^{l}$ and $\beta_{R\,i}^{l}$, respectively, denote an item-specific sender effect and an item-specific receiver effect. Then, the dyadic format of Model 1 and Model 2 can be, respectively, simplified as:


(10) $$y_{i\,S\,R}^{l}=\beta_{0}+\beta_{S\,i}^{l}\,\theta_{S}^{l}+\beta_{R\,i}^{l}\,\theta_{R}^{l}+\xi_{i}^{l}$$

(11) $$y_{i\,S\,R}^{l}=\beta_{0}+\beta_{S\,i}^{l}\,\theta_{S}^{l}+\beta_{R\,i}^{l}\,\theta_{R}^{l}+\beta_{D\,i}^{l}\,E_{d}+\xi_{i}^{l}.$$

The item-specific parameters, $\beta_{S\,i}^{l}$, $\beta_{R\,i}^{l}$, and $\beta_{D\,i}^{l}$, describe the characteristics of a given item—namely, its ability to differentiate rating senders’ traits, rating receivers’ traits, and sender–receiver trait dissimilarities.

Equations (10) and (11) specify the relationships between the observed item responses and the latent traits, and therefore they are regarded as the measurement model portion of the LIDM. Another key component is the structural model, which specifies the relationships among all latent traits within a dyad. Let **Θ**_S*R*_ be an *L*×2 matrix, containing the latent trait scores for a dyad. Then, the structural model can be expressed as:


(12) $$\Theta_{S\,R}=\mu_{S\,R}+\Xi_{S\,R}$$

where μ_**S***R*_ is an *L*×2 matrix containing the means for all latent traits, and **Ξ**_S*R*_ is an *L*×2 matrix containing the random errors of all latent traits. Let **Ξ** be either of column vectors of **Ξ**_S*R*_, such that **Ξ** follows a multivariate normal distribution 𝒩(**0**,**Σ**_θ_).

Under the LIDM, the probability of observing the relational data for a social network with *n* members generated from Model 1 can be written as:


(13) $$P\left(\text{Y}|\beta_{0},{\text{B}}_{S},{\text{B}}_{R},\Theta \right)=\prod_{S=1}^{n}\prod_{R=1}^{n}\prod_{l=1}^{L}\prod_{i=1}^{I}P\,\left(\text{v1}|\beta_{0},\beta_{S\,i}^{l},\beta_{R\,i}^{l},\theta_{S}^{l},\theta_{R}^{l}\right)\times \prod_{S=1}^{n}\prod_{R=1}^{n}\prod_{l=1}^{L}\prod_{i=1}^{I}P\,\left(\text{v2}|\beta_{0},\beta_{S\,i}^{l},\beta_{R\,i}^{l},\theta_{S}^{l},\theta_{R}^{l}\right),$$

where **B_S_** and **B_R_** are *T* × 1 matrices, *T* = *I* × *L*, containing all item-specific sender effects and receiver effects, respectively. Also, **Θ** is a *W*×1 matrix, such that *W* = *n*×*L*, containing the latent trait scores for all group members. The probability of observing relational data for a social network with *n* members generated from Model 2 can be written as:


(14) $$P\left(\text{Y}|\beta_{0},{\text{B}}_{S},{\text{B}}_{R},{\text{B}}_{D},\Theta \right)=\prod_{S=1}^{n}\prod_{R=1}^{n}\prod_{l=1}^{L}\prod_{i=1}^{I}P\,\left(\text{v1}|\beta_{0},\beta_{S\,i}^{l},\beta_{R\,i}^{l},\beta_{D\,i}^{l},\theta_{S}^{l},\theta_{R}^{l}\right)\times \prod_{S=1}^{n}\prod_{R=1}^{n}\prod_{l=1}^{L}\prod_{i=1}^{I}P\,\left(\text{v2}|\beta_{0},\beta_{S\,i}^{l},\beta_{R\,i}^{l},\beta_{D\,i}^{l},\theta_{S}^{l},\theta_{R}^{l}\right),$$

where **B_D_** is a *T* × 1 matrix containing the distance effects associated with all items.

According to the LIDM, the prior example of interpersonal trust data can be modeled as follows. Under Model 1, the response of student A to an ABT item regarding student B, is determined by B’s ABT trait score and A’s own ABT trait score. Similarly, under Model 2, A’s response to an ABT item is determined by their own and B’s ABT scores, as well as their relative position (i.e., the distance) in a latent space defined by two given dimensions—the ABT trait and the CBT trait. The closer two students are in this latent “trust” space, the more likely they can be regarded as similar in considering their “trust” traits. Students of the same “trust” type may share some common values and/or other important common characteristics. In the LIDM, as a testable hypothesis in explaining one’s response to any items, it is possible these similarities may have a unique contribution to the responses (beyond that accounted for by both students’ traits being measured by any given “trust” item). The same pattern would hold for the students’ responses to the CBT items. The models produce the posterior distributions of all students’ ABT and CBT latent trait scores, as well as the covariance between these two latent traits; these distributions can be summarized with means (i.e., the estimates *via* expected *a posteriori* [EAP] estimation) or medians. Also, the weights of all directed connections among students along the ABT and CBT dimensions can be derived based on the estimates of the latent traits and their related effects and interpreted using the original metric of the survey.

### Parameter Estimation

To estimate the LIDM, we have taken a Bayesian approach, in which the objective is to find the desired estimate using the posterior probability distribution of a given parameter. As for the prior distributions of model parameters, we assume:


$$\,\beta_{0}∼N\,\left(\mu_{0},\sigma_{0}\right),$$

$$\,\xi_{i}^{l}∼N\,\left(0,\sigma_{i}^{l}\right),$$

$${\text{B}}_{S\,R\,i}^{l}=\left(\beta_{S\,i}^{l},\beta_{R\,i}^{l}\right)∼N\,\left(\mu_{b},\Sigma_{b}\right)\,w\,i\,t\,h\,\beta_{S\,i}^{l}\in \left(-\infty ,+\infty \right)$$

$$\,a\,n\,d\,\beta_{R\,i}^{l}\in \left(0,+\infty \right),$$

$$\,\beta_{D\,i}^{l,*}∼L\,o\,g\,n\,o\,r\,m\,a\,l\,\left(\mu_{D},\sigma_{D}\right),$$

$$\,\Theta_{S}\,a\,n\,d\,\Theta_{R}∼N\,\left(\mu_{\theta },\Sigma_{\theta }\right),$$

where $\beta_{D\,i}^{l,*}$ = $-\beta_{D\,i}^{l}$, and ${\text{B}}_{S\,R\,i}^{l}$ is a vector of the sender effect and receiver effect associated with the same item, which follows a truncated multivariate normal distribution in which the dimension of receiver effect is bounded above 0. Then, the full posterior distribution of the parameters in Model 1 and Model 2, respectively, can be defined as:


(15) $$P\left(\beta_{0},{\text{B}}_{S},{\text{B}}_{R},\Theta_{S\,R},\sigma |\text{Y}\right)\propto P\left(\text{Y}|\beta_{0},{\text{B}}_{S},{\text{B}}_{R},\Theta_{S\,R},\sigma \right)*P\left(\beta_{0}^{l}\right)*\prod_{S=1}^{n}\prod_{R=1}^{n}\prod_{l=1}^{L}\left[P\left(\theta_{S}^{l}\right)*P\left(\theta_{R}^{l}\right)\right]*\prod_{l=1}^{L}\prod_{i=1}^{I}\left[P\,\left({\text{B}}_{S\,R\,i}^{l}\right)*P\,\left(\sigma_{i}^{l}\right)\right],$$

and


$$P\left(\beta_{0},{\text{B}}_{S},{\text{B}}_{R},{\text{B}}_{D},\Theta_{S\,R},\sigma |\text{Y}\right)\propto P\left(\text{Y}|\beta_{0},{\text{B}}_{S},{\text{B}}_{R},{\text{B}}_{D},\Theta_{S\,R},\sigma \right)*P\left(\beta_{0}^{l}\right)*\prod_{S=1}^{n}\prod_{R=1}^{n}\prod_{l=1}^{L}\left[P\left(\theta_{S}^{l}\right)*P\left(\theta_{R}^{l}\right)\right]*$$

(16) $$\prod_{l=1}^{L}\prod_{i=1}^{I}\left[P\,\left({\text{B}}_{S\,R\,i}^{l}\right)*P\,\left(\beta_{D\,i}^{l}\right)*P\,\left(\sigma_{i}^{l}\right)\right].$$

The likelihood function in the Functions (15) and (16) can be expanded as in Equations (13) and (14).

We used a Markov chain Monte Carlo (MCMC) to sample multivariate random quantities from a full posterior distribution. In this study, sampling from the posterior distributions of parameters of interest was implemented by the computer program JAGS (Just Another Gibbs Sampler, Plummer, 2003) *via* a slice sampling algorithm (Neal, 2003). The point estimates of model parameters have been summarized by the EAP estimates of posterior distributions.

## A Simulation Study

### Design

The primary goal of this simulation study reported next was to evaluate how accurately the parameters of two proposed LIDM can be recovered. To do so, psychometric relational data were simulated under Model 1 and Model 2 and the parameters of each model were estimated with its own data. The accuracy of parameter recovery was evaluated by root-mean-squared errors (*RMSE*), normalized root-mean-squared error (*NRMSE*), bias, and coefficient of determination (*R*^2^), which are, respectively, calculated as follows:


(17) $$R\,M\,S\,E=\sqrt{\frac{\sum_{i=1}^{n}{\left({\hat{\varphi }}_{i}-\varphi_{i}\right)}^{2}}{n}},$$

(18) $$N\,R\,M\,S\,E=\sqrt{\frac{\sum_{i=1}^{n}{\left({\hat{\varphi }}_{i}-\varphi_{i}\right)}^{2}}{n}}/\sigma_{\varphi },$$

(19) $$B\,i\,a\,s=\frac{\sum_{i=1}^{n}\left({\hat{\varphi }}_{i}-\varphi_{i}\right)}{n},$$

and


(20) $$R^{2}=1-\frac{\sum_{i=1}^{n}{\left({\hat{\varphi }}_{i}-\varphi_{i}\right)}^{2}}{\sum_{i=1}^{n}{\left(\varphi_{i}-\bar{\varphi }\right)}^{2}},$$

where *n* denotes the number of simulated data sets, φ_*i*_ and ${\hat{\varphi }}_{i}$ denote (respectively) the true value of a given parameter φ and its estimated value in each simulation and estimation trial, and $\bar{\varphi }$ denotes the mean of the true value of φ across *n* simulations. In Function (18), σ_φ_ represents the standard deviation of $\hat{\varphi }{}_{i}$.

The second goal of this study was to evaluate the impact of network size on the efficacy of model estimation. To that end, the performance of each model was evaluated with varying network sizes in terms of its accuracy in recovering its own model parameters with the change of network size. Lastly, we examined the accuracy of parameter recovery when each model was estimated with data generated under the other model—this was done to evaluate the robustness of parameter estimates to a particular mis-specified model under the framework of LIDM.

To evaluate the impact of network size on the efficacy of model estimation, five network sizes (*n* = 5, 10, 20, 30, and 100) were investigated. These network sizes were chosen to reflect a research context in which eight items were administered to measure a two-dimensional relational construct. In such a scenario, each group member rated all others on all eight items.

### Data Generation and Evaluation

Using a simulated network size of 5, 10, 20, 30, or 100, dyadic response data were generated based on the parameterizations of the two proposed models from eight items; each of the two latent dimensions was measured by four items and each item only measured one dimension. Table 2 shows the model parameterizations and the probability distributions used to generate each model’s parameters. The probability distributions were chosen based on the properties of each parameter, but it was expected that, regardless of choice of prior distributions, the estimation procedures would recover all model parameters accurately. The means and standard deviations of the simulated (true) values for all parameters are shown in Table 3.

**TABLE 2:** Model specifications and parameter settings for data generation.

Models	Specification	Parameters and Distributions
Model 1	$y_{i\,S\,R}^{l}=\beta_{0}^{l}+\beta_{S\,i}^{l}\,\theta_{S}^{l}+\beta_{R\,i}^{l}\,\theta_{R}^{l}+\xi_{i}^{l}$	$\beta_{0}^{l}$∼𝒩(0, 1) $\beta_{R\,i}^{l}$∼𝒩(μ_*R*_,σ_*R*_)(0) ${\text{β}}_{Si}^{'}\sim N\left({\text{μ}}_{S}{\text{ρ}}_{b}*(\sigma_{S}/\sigma_{R}\right)*({\text{β}}_{Ri}^{'}-{\text{μ}}_{R}),\sigma_{S}*\sqrt{1}-{\text{ρ}}_{b}^{2})$ μ_*S*_ = μ_*R*_ = 0 σ_*S*_ = σ_*R*_ = 1 ρ_β_∼ *U* (0.1, 0.3) $\Theta_{SR}\sim N\left(\left[\begin{array}{c} 0\\ 0 \end{array}\right],\left[\begin{array}{cc} 1 & \rho_{\theta }\;\\ \rho_{\theta }\; & 1 \end{array}\right]\right)$ ρ_θ_∼ *U* (0.2, 0.3) $\xi_{i}^{l}$∼𝒩(0, 1)
Model 2	$y_{iSR}^{l}{= \beta }_{0}^{l}\;\beta_{Si}^{l}\theta_{S}^{l}\;\beta_{Ri}^{l}\theta_{R}^{l}\beta_{Di}^{l}E_{d}\xi_{i}^{l}\;$	$\beta_{D\,i}^{l}$∼ *U* (–0.4,0) All other parameters use the same settings as their counterparts in Model 1.

**TABLE 3:** Means and standard deviations of simulated values for model parameters.

	Network Size (NS)									
	NS = 5		NS = 10		NS = 20		NS = 30		NS* = 100	
Parameters	*Mean*	*SD*	*Mean*	*SD*	*Mean*	*SD*	*Mean*	*SD*	*Mean*	*SD*
β_0_ (Grand Mean)	0.04 (0.01)	1.01 (1.03)	0.02 (0.02)	1.00 (1.02)	0.08 (0.05)	1.01 (0.97)	−0.09 (0.01)	1.04 (0.98)	0.00 (0.00)	1.00 (1.00)
B_**S**_ (Sender Effect)	−0.03 (0.04)	1.07 (0.99)	−0.02 (0.09)	1.02 (1.00)	0.00 (0.03)	1.04 (0.99)	−0.02 (0.01)	1.00 (0.98)	0.00 (0.00)	1.01 (1.02)
B_**R**_ (Receiver Effect)	0.23 (0.19)	0.96 (1.00)	0.25 (0.19)	0.99 (0.95)	0.24 (0.23)	1.00 (0.98)	0.24 (0.20)	0.98 (0.97)	0.21 (0.23)	1.00 (0.99)
ρ_β_ (Correlation between β_*Si*_ and β_*Ri*_)	0.19 (0.23)	0.06 (0.05)	0.19 (0.19)	0.03 (0.02)	0.23 (0.21)	0.04 (0.06)	0.20 (0.20)	0.06 (0.02)	0.24 (0.20)	0.02 (0.03)
**Θ** (Trait Score)	0.04 (0.02)	0.98 (1.04)	0.02 (0.05)	1.01 (1.00)	−0.01 (0.00)	1.01 (0.99)	0.02 (−0.02)	1.00 (1.00)	0.00 (0.01)	1.01 (1.02)
ρ_θ_ (Trait Correlation)	0.23 (0.25)	0.04 (0.04)	0.25 (0.22)	0.02 (0.01)	0.27 (0.23)	0.05 (0.03)	0.22 (0.24)	0.06 (0.02)	0.25 (0.25)	0.04 (0.02)
$\xi_{i}^{l}$ (Residuals)	0.02 (0.04)	1.03 (1.06)	0.01 (0.00)	1.01 (1.01)	0.02 (0.03)	1.00 (1.02)	0.01 (0.00)	1.00 (0.99)	0.01 (0.00)	1.00 (1.00)
B_**D**_ (Effect of Distance Metric)	−0.20	0.10	−0.20	0.11	−0.21	0.11	−0.21	0.12	−0.20	0.12

*The number of simulated data set is 100 for a network size of 100 instead of 300. Results from Model 2 are presented in parentheses.

Accuracy of model parameter recovery was indicated by *RMSE*, *NRMSE*, bias, and *R*^2^. The calculations of all these indexes were based on 300 simulated data sets for all investigated network sizes except for size 100, which were based on 100 replications instead due to the inefficiency of computation for this condition.

### Data Analysis

Bayesian estimation was performed using the program JAGS (Just Another Gibbs Sampler, Plummer, 2003). For all models, two Markov chains were generated with each chain including 100,000 samples (number of iterations = 100,000). To represent the posterior distribution of each parameter, the first 50,000 samples were discarded as a burn-in period. Also, to lower the autocorrelation in a single Markov chain, every 2nd simulated sample (rate of thinning = 2) was kept in the chain. In addition, given the complexity of model parameterization, 10,000 adaptive steps were allowed for the program to adjust its algorithms (i.e., change its step size for random walks) to different model parameters. Table 4 shows the prior distributions used for all parameters.

**TABLE 4:** Prior distributions for model parameters.

Parameters	Prior Distributions
β_0_ (Grand Mean)	β_0_∼*N* (0, 10)
β_*Ri*_ (Receiver Effect)	β_*Ri*_∼*N*(μ_β_*R*__,σ_β_*R*__)*T*(0)
β_*Si*_ (Sender Effect)	$\beta_{S\,i}∼N\,\left(\mu_{\beta_{S}}+\lambda *\left(\beta_{R\,i}-\mu_{R}\right),\,\sigma_{\beta_{R}}*\sqrt{1-\rho_{b}^{2}}\right)$
μ_β_*R*__	μ_β_*R*__=0
μ_β_*S*__	μ_β_*S*__=0
λ	λ∼*N*(0,1000)
σ_β_*R*__	σ_β_*R*__=1
σ_β_*S*__	σ_β_*S*__∼*U*(1,1000)
ρ_β_(Correlation between β_*Si*_ and β_*Ri*_)	$\rho_{\beta }=\lambda *\left(\frac{\sigma_{\beta_{R}}}{\sigma_{\beta_{S}}}\right)$
θ_*SR*_ (Trait Score)	$\theta_{SR}\sim N\left(\left[\begin{array}{c} 0\\ 0 \end{array}\right],\left[\begin{array}{cc} 1 & \rho_{\theta }\;\\ \rho_{\theta }\; & 1 \end{array}\right]\right)$
ρ_θ_ (Trait Correlation)	ρ_θ_∼*U* (0, 1)
τ () (Variance of Errors)	τ∼*Gamma* (0.01,0.01)
βD⁢i* (Effect of the Distance Metric, βD⁢i* = –β_*Di*_)	βD⁢i*∼L⁢o⁢g⁢n⁢o⁢r⁢m⁢a⁢l⁢(0,10)

## Results

### The Effectiveness of Model Parameterization and Model Estimation

Table 5 presents *RMSE*, *NRMSE*, bias, *R*^2^, and the cross-sample average of the Gelman-Rubin statistic ($\hat{R}$) for each parameter. Overall, the chains for all samples converged successfully, yielding estimates^1^ for all parameters with small biases and high *R*^2^ values. These convergence and parameter recovery results support the parameterizations of proposed LIDM. As shown in Table 5, *RMSE*, *NRMSE*, and bias under most conditions were small and all *R*^2^ values were above 0.95, suggesting the estimation procedures produced accurate estimates.

**TABLE 5:** Root-mean-squared error (*RMSE*), normalized root-mean-squared error (*NRMSE*), bias, and coefficient of determination (*R*^2^) of parameter recovery, convergence diagnosis index ($\hat{R})$, and effective sample size (*ESS*).

	Network Size (NS)											
	NS = 5					NS = 10						
Parameters	*RMSE*	*NRMSE*	*Bias*	*R* ^2^	Mean of $\hat{R}$	*ESS*	*RMSE*	*NRMSE*	*Bias*	*R* ^2^	Mean of $\hat{R}$	*ESS*
β_0_	0.150 (0.190)	0.149 (0.184)	0.030 (0.020)	0.952 (0.950)	1.00 (1.00)	21104 (13721)	0.150 (0.180)	0.150 (0.176)	0.010 (0.020)	0.954 (0.953)	1.00 (1.00)	31120 (16741)
**B_S_**	0.190 (0.260)	0.178 (0.263)	0.060 (0.030)	0.956 (0.953)	1.00 (1.00)	10497 (5214)	0.210 (0.250)	0.206 (0.250)	0.040 (0.070)	0.957 (0.958)	1.00 (1.00)	19022 (10225)
**B_R_**	0.160 (0.160)	0.167 (0.160)	0.010 (0.008)	0.953 (0.957)	1.00 (1.00)	13880 (4901)	0.100 (0.120)	0.101 (0.126)	0.005 (0.010)	0.961 (0.958)	1.00 (1.00)	6921 (5303)
ρ_β_	0.200 (0.220)	3.333 (4.400)	0.009 (0.010)	0.956 (0.956)	1.00 (1.00)	10241 (4009)	0.150 (0.150)	5.000 (7.500)	0.010 (0.009)	0.959 (0.960)	1.00 (1.00)	7449 (4373)
**B_D_**	0.210	2.100	0.020	0.950	1.00	5290	0.200	1.818	0.030	0.954	1.00	3772
**Θ**	0.310 (0.300)	0.316 (0.288)	−0.020 (−0.010)	0.952 (0.954)	1.00 (1.00)	11932 (4233)	0.300 (0.280)	0.297 (0.280)	−0.010 (−0.007)	0.956 (0.953)	1.00 (1.00)	7288 (5332)
ρ_θ_	0.100 (0.100)	2.500 (2.500)	0.020 (0.020)	0.950 (0.952)	1.00 (1.00)	9236 (3475)	0.110 (0.090)	5.500 (9.000)	0.030 (0.030)	0.957 (0.957)	1.00 (1.00)	6421 (3079)
σ	0.120 (0.110)	0.117 (0.104)	−0.020 (−0.040)	0.953 (0.955)	1.00 (1.00)	47966 (41209)	0.120 (0.150)	0.119 (0.149)	−0.040 (−0.070)	0.955 (0.955)	1.00 (1.00)	40291 (47330)

	**Network Size**											
	**NS = 20**					**NS = 30**						
**Parameters**	***RMSE***	***NRMSE***	***Bias***	*R* ^2^	**Mean of $\hat{R}$**	***ESS***	***RMSE***	***NRMSE***	***Bias***	***R* ^2^**	**Mean of $\hat{R}$**	***ESS***

β_0_	0.070 (0.10)	0.069 (0.103)	0.008 (0.011)	0.973 (0.973)	1.00 (1.00)	33214 (14981)	0.030 (0.050)	0.029 (0.051)	−0.009 (0.007)	0.991 (0.991)	1.00 (1.00)	4301 (4019)
**B_S_**	0.130 (0.130)	0.125 (0.131)	0.040 (0.050)	0.971 (0.973)	1.00 (1.00)	29481 (13874)	0.080 (0.100)	0.080 (0.102)	0.010 (0.020)	0.993 (0.993)	1.00 (1.00)	6004 (3891)
**B_R_**	0.070 (0.060)	0.070 (0.061)	0.010 (0.007)	0.968 (0.971)	1.00 (1.00)	29672 (18518)	0.030 (0.040)	0.031 (0.041)	0.006 (0.006)	0.991 (0.992)	1.00 (1.00)	6442 (4701)
ρ_β_	0.100 (0.090)	2.500 (1.500)	0.008 (0.010)	0.970 (0.972)	1.00 (1.00)	20133 (11339)	0.050 (0.060)	0.833 (3.000)	0.004 (0.007)	0.990 (0.987)	1.00 (1.00)	3180 (3127)
**B_D_**	0.060	0.545	0.003	0.975	1.00	7812	0.050	0.417	0.004	0.994	1.00	2903
**Θ**	0.190 (0.190)	0.188 (0.192)	−0.007 (0.004)	0.967 (0.967)	1.00 (1.00)	10213 (7726)	0.090 (0.120)	0.090 (0.120)	−0.004 (0.001)	0.993 (0.991)	1.00 (1.00)	4712 (4004)
ρ_θ_	0.120 (0.140)	2.400 (4.667)	0.060 (0.060)	0.969 (0.967)	1.00 (1.00)	20113 (10027)	0.080 (0.120)	1.333 (6.000)	−0.090 (−0.070)	0.992 (0.992)	1.00 (1.00)	5711 (3870)
σ	0.080 (0.080)	0.080 (0.078)	−0.010 (−0.010)	0.971 (0.973)	1.00 (1.00)	48910 (49013)	0.040 (0.050)	0.040 (0.051)	−0.008 (−0.008)	0.993 (0.994)	1.00 (1.00)	39244 (33278)

		**Network Size**											
		NS* = 100											
**Parameters**	***RMSE***			***NRMSE***	***Bias***			***R* ^2^**	**Mean of $\hat{R}$**			***ESS***

β_0_	0.009 (0.006)			0.009 (0.006)	0.007 (0.002)			0.996 (0.997)	1.00 (1.00)			5014 (3772)
**B_S_**	0.010 (0.008)			0.010 (0.008)	0.003 (0.008)			0.995 (0.992)	1.00 (1.00)			5373 (2499)
**B_R_**	0.006 (0.010)			0.006 (0.010)	0.009 (0.004)			0.993 (0.995)	1.00 (1.00)			4702 (3317)
ρ_β_	0.010 (0.009)			0.500 (0.300)	0.002 (0.002)			0.997 (0.994)	1.00 (1.00)			2978 (2470)
B_**D**_	0.010			0.083	0.001			0.992	1.00			2144
**Θ**	0.030 (0.030)			0.030 (0.029)	0.002 (0.006)			0.996 (0.998)	1.00 (1.00)			3954 (3077)
ρ_θ_	0.020 (0.020)			0.500 (1.00)	−0.010 (0.007)			0.997 (0.995)	1.00 (1.00)			4292 (3113)
σ	0.005 (0.007)			0.005 (0.007)	0.000 (−0.001)			0.997 (0.997)	1.00 (1.00)			45330 (40291)

*The number of simulations is 100 for a network size of 100 and 300 otherwise. Results from Model 2 are presented in parentheses. β_0_ = the grand mean; **B_S_** = the matrix for sender effects; **B_R_** = the matrix for receiver effect; ρ_β_ = the correlation coefficient between sender effect and receiver effect; **B_D_** = the matrix for effect of distance metric; **Θ** the matrix for latent trait scores; ρ_θ_ = the correlation coefficient between two latent traits; σ = the matrix for variance of errors; ESS = effective sample size.

The accuracy of recovery varied across parameters and conditions. In estimating both models, the largest *RMSE* (0.31 for Model 1 and 0.30 for Model 2) was associated with the latent trait matrix (**Θ**) when the network size was 5, although the biases associated with **Θ** were small under all conditions. Also, in fitting Model 2, the *RMSE* and bias associated with the effect of the distance metric (B_**D**_) were small. In estimating both models, relatively large *NRMSE*s were observed for the parameters ρ_β_ and ρ_θ_ under all conditions due to the relatively small standard deviations of their simulated values. Also, Model 2 produced larger *NRMSE*s under most conditions than did Model 1 for almost all parameters, suggesting Model 1 generally resulted in more accurate estimates than did Model 2.

### The Effects of Network Size on Model Estimation

As shown in Table 5, for most parameters in both models, clearly smaller *RMSE* and bias values, as well as higher *R*^2^ values, were observed as network size grew, suggesting the overall accuracy of model estimation improved when estimating both models with a larger network. An exception was the correlation coefficient ρ_θ_. The changes of *RMSE* and bias associated with ρ_θ_ as a function of network size were not as clear. Specifically, the largest *RMSE* for ρ_θ_ was associated with a network size of 20 in fitting both models. It should be noted that although the size of the matrix **Θ** was a function of network size, smaller *RMSE* and bias were observed for **Θ** as the network grew.

### The Robustness of Model Estimation to the Violation of Model Parametrization

Table 6 shows the indices for parameter recovery accuracy and MCMC convergence when fitting one proposed model with data generated under the other model. In analyzing data that violate model parameterizations, both models converged successfully with all simulated samples. Moreover, both models produced small biases for the latent trait matrix (**Θ**) with a wide range spanning from 0.002 to 0.020. *RMSE* ranged from 0.01 to 0.35, with larger errors arising from smaller networks. The recovery of correlation coefficient (ρ_θ_) yielded generally small *RMSE* (ranging from 0.03 to 0.21) and small but also widely ranging bias (spanning from 0.002 to 0.030). Moreover, high *R*^2^ (above 0.95) was observed for all parameters under almost all conditions. These results support the robustness of both models despite misspecification.

**TABLE 6:** Root-mean-squared error (*RMSE*), normalized root-mean-squared error (*NRMSE*), bias, and coefficient of determination (*R*^2^) of parameter recovery, convergence diagnosis index ($\hat{R})$, and effective sample size (*ESS*) from model cross-estimation.

	Network Size (NS)											
	NS = 5					NS = 10						
Parameters	*RMSE*	*NRMSE*	*Bias*	*R* ^2^	Mean of $\hat{R}$	*ESS*	*RMSE*	*NRMSE*	*Bias*	*R* ^2^	Mean of $\hat{R}$	*ESS*
**Θ**	0.350 (0.300)	0.357 (0.288)	0.020 (0.020)	0.949 (0.953)	1.00 (1.00)	10326 (6029)	0.350 (0.320)	0.347 (0.320)	0.010 (−0.020)	0.958 (0.955)	1.00 (1.00)	11219 (4134)
ρ_θ_	0.210 (0.150)	5.250 (3.750)	0.030 (0.030)	0.955 (0.954)	1.00 (1.00)	12193 (4670)	0.150 (0.120)	1.500 (12.000)	0.030 (0.030)	0.961 (0.955)	1.00 (1.00)	9233 (3021)

	**Network Size**											
	**NS = 20**					**NS = 30**						
**Parameters**	***RMSE***	***NRMSE***	***Bias***	***R* ^2^**	**Mean of $\hat{R}$**	***ESS***	***RMSE***	***NRMSE***	***Bias***	***R* ^2^**	**Mean of $\hat{R}$**	***ESS***

**Θ**	0.310 (0.270)	0.307 (0.273)	0.008 (0.020)	0.972 (0.972)	1.00 (1.00)	10311 (3498)	0.200 (0.130)	0.200 (0.130)	−0.002 (−0.002)	0.991 (0.995)	1.00 (1.00)	11012 (2981)
ρ_θ_	0.140 (0.140)	2.800 (4.667)	−0.007 (−0.020)	0.968 (0.971)	1.00 (1.00)	8231 (2398)	0.100 (0.090)	1.667 (4.500)	−0.010 (0.008)	0.993 (0.993)	1.00 (1.00)	6348 (3025)

		**Network Size**												
		NS* = 100												
**Parameters**	***RMSE***			***NRMSE***	***Bias***			***R* ^2^**	**Mean of $\hat{R}$**			***ESS***

**Θ**	0.010 (0.008)			0.010 (0.008)	0.003 (0.007)			0.994 (0.996)	1.00 (1.00)			4920 (2914)
ρ_θ_	0.030 (0.030)			0.750 (1.500)	−0.012 (0.005)			0.995 (0.997)	1.00 (1.00)			5049 (3622)

*The number of simulations is 100 for a network size of 100 and 300 otherwise. Results from Model 2 are presented in parentheses. **Θ** the matrix for latent trait scores; ρ_θ_ = the correlation coefficient between two latent traits; ESS = effective sample size.

## Fitting the Latent Interdependence Models on Empirical Data

**FIGURE 1:**
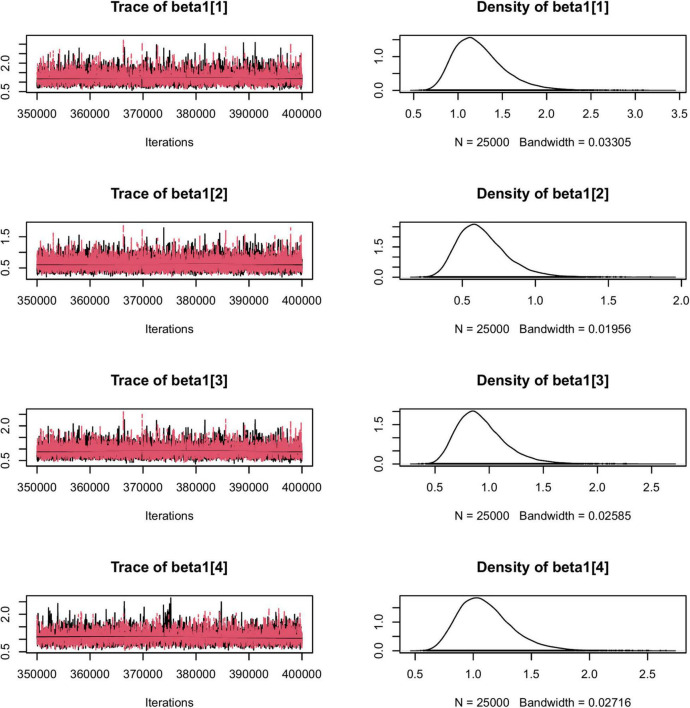
The traces for model convergence and the posterior distributions of a portion of Model 1 parameters.

**FIGURE 2:**
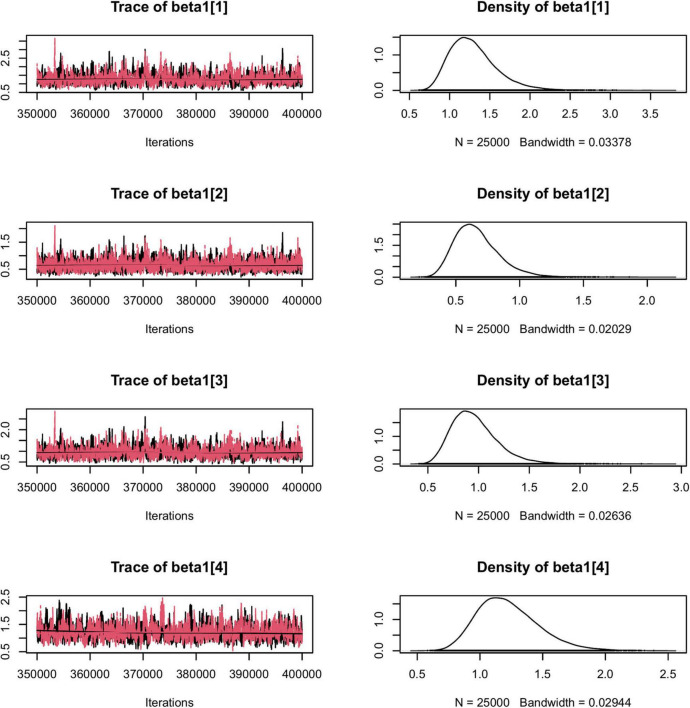
The traces for model convergence and the posterior distributions of a portion of Model 2 parameters.

To illustrate the usefulness of the LIDM, we estimated the two proposed models using interpersonal trust survey data as described previously. The data are complete item responses from a total of 105 dyads. Table 7 presents the results of the two proposed models with the interpersonal trust data. Due to limited space, in Figures 1, 2 we present only some examples of the traces for model convergence and the posterior distributions of model parameters; a complete report of the parameter estimation results is included in the Supplementary Materials, which can be accessed *via* the attached FigShare link. Overall, the two models produced close estimates for most parameters. Besides producing the estimates for each student’s trait scores for affect-based trust (ABT) and cognition-based trust (CBT), the models also provided estimates for the items’ differentiation of dyad members’ traits and the dissimilarity between traits. Specifically, large positive item-specific sender effects (ranging from 0.64 to 1.29 under Model 1 and from 0.67 to 1.39 under Model 2) were found for all six items, indicating all items were highly sensitive to the change of rating sender’s trait scores. That is, students with different trust scores tend to respond quite differently to these items regarding a same rating target.

**TABLE 7:** Means and standard deviations of LIDM estimated parameters, convergence diagnosis index ($\hat{R})$, and effective sample size (*ESS*) in empirical data analysis.

	Model 1					Model 2				
Parameters	*Mean*	*SD*	*Median*	$\hat{R}$	*ESS*	*Mean*	*SD*	*Median*	$\hat{R}$	*ESS*
B_**S**_ [1]	1.24	0.29	1.20	1.00	50000	1.30	0.30	1.26	1.00	6817
B_**S**_ [2]	0.64	0.17	0.62	1.00	15203	0.67	0.18	0.64	1.00	4365
B_**S**_ [3]	0.93	0.23	0.90	1.00	50000	0.96	0.23	0.93	1.00	10029
B_**S**_ [4]	1.11	0.23	1.08	1.00	1563	1.22	0.25	1.20	1.00	1377
B_**S**_ [5]	1.29	0.26	1.25	1.00	1923	1.39	0.27	1.35	1.00	1208
B_**S**_ [6]	0.87	0.19	0.85	1.00	2679	0.95	0.20	0.92	1.00	1070
B_**R**_ [1]	0.14	0.08	0.13	1.00	15475	0.15	0.09	0.13	1.00	6971
B_**R**_ [2]	0.22	0.10	0.21	1.00	50000	0.23	0.11	0.22	1.00	6875
B_**R**_ [3]	0.08	0.06	0.07	1.00	22145	0.09	0.07	0.07	1.00	43137
B_**R**_ [4]	0.57	0.14	0.55	1.00	2405	0.65	0.15	0.63	1.00	2129
B_**R**_ [5]	0.23	0.09	0.22	1.00	32798	0.25	0.10	0.24	1.00	3142
B_**R**_ [6]	0.68	0.16	0.66	1.00	2100	0.74	0.16	0.72	1.00	1913
B_**D**_ [1]	−	−		−	−	−0.02	0.03	0.01	1.00	1766
B_**D**_ [2]	−	−		−	−	−0.03	0.03	0.02	1.00	3866
B_**D**_ [3]	−	−		−	−	−0.02	0.02	0.01	1.00	11370
B_**D**_ [4]	−	−		−	−	−0.09	0.06	0.08	1.00	4771
B_**D**_ [5]	−	−		−	−	−0.03	0.03	0.02	1.00	19060
B_**D**_ [6]	−	−		−	−	−0.03	0.04	0.02	1.00	12175
**Θ** [1,1]	0.09	0.22	0.08	1.00	3321	0.09	0.22	0.09	1.00	829
**Θ** [2,1]	0.52	0.25	0.50	1.00	14905	0.49	0.24	0.48	1.00	2330
**Θ** [3,1]	0.14	0.23	0.14	1.00	5322	0.14	0.22	0.14	1.00	2420
**Θ** [4,1]	−1.12	0.31	−1.10	1.00	3663	−1.05	0.31	−1.03	1.00	770
**Θ** [5,1]	0.53	0.25	0.51	1.00	6228	0.54	0.25	0.52	1.00	1156
**Θ** [6,1]	−0.99	0.29	−0.96	1.00	4474	−0.92	0.29	−0.90	1.00	922
**Θ** [7,1]	−0.76	0.26	−0.74	1.00	4084	−0.71	0.27	−0.70	1.00	847
**Θ** [8,1]	1.25	0.34	1.22	1.00	6936	1.25	0.33	1.22	1.00	2254
**Θ** [9,1]	−0.22	0.22	−0.21	1.00	5114	−0.19	0.23	−0.19	1.00	1031
**Θ** [10,1]	1.18	0.33	1.16	1.00	9700	1.16	0.32	1.13	1.00	4096
**Θ** [11,1]	−0.12	0.22	−0.12	1.00	5643	−0.11	0.22	−0.10	1.00	636
**Θ** [12,1]	−1.22	0.33	−1.20	1.00	4838	−1.14	0.32	−1.12	1.00	1514
**Θ** [13,1]	−1.01	0.30	−0.99	1.00	4309	−0.93	0.29	−0.91	1.00	605
**Θ** [14,1]	0.76	0.27	0.74	1.00	8907	0.75	0.26	0.73	1.00	1866
**Θ** [15,1]	0.43	0.25	0.42	1.00	10089	0.43	0.24	0.42	1.00	1329
**Θ** [1,2]	−0.50	0.20	−0.49	1.00	14425	−0.44	0.18	−0.43	1.00	488
**Θ** [2,2]	0.05	0.18	0.05	1.00	3895	0.07	0.17	0.07	1.00	889
**Θ** [3,2]	−0.41	0.19	−0.40	1.00	29616	−0.36	0.18	−0.35	1.00	1144
**Θ** [4,2]	0.28	0.18	0.27	1.00	2785	0.30	0.18	0.29	1.00	8410
**Θ** [5,2]	−0.18	0.18	−0.18	1.00	7136	−0.14	0.17	−0.13	1.00	941
**Θ** [6,2]	0.08	0.18	0.08	1.00	3242	0.12	0.17	0.11	1.00	1437
**Θ** [7,2]	−0.60	0.21	−0.59	1.00	50000	−0.53	0.18	−0.51	1.00	811
**Θ** [8,2]	2.12	0.43	2.09	1.00	2404	2.04	0.42	2.01	1.00	2902
**Θ** [9,2]	−1.12	0.27	−1.10	1.00	50000	−0.98	0.23	−0.96	1.00	739
**Θ** [10,2]	0.03	0.17	0.03	1.00	3100	0.07	0.17	0.07	1.00	5451
**Θ** [11,2]	−0.45	0.19	−0.44	1.00	32028	−0.39	0.18	−0.38	1.00	933
**Θ** [12,2]	−1.01	0.26	−0.99	1.00	50000	−0.87	0.23	−0.85	1.00	421
**Θ** [13,2]	1.38	0.30	1.36	1.00	1931	1.33	0.30	1.30	1.00	9637
**Θ** [14,2]	−0.30	0.19	−0.29	1.00	17123	−0.24	0.17	−0.24	1.00	1131
**Θ** [15,2]	1.73	0.36	1.71	1.00	2894	1.65	0.35	1.62	1.00	9236
ρ_θ_	0.41	0.24	0.38	1.00	50000	0.41	0.25	0.39	1.00	7619
ρ_β_	0.81	0.29	0.92	1.00	50000	0.81	0.29	0.91	1.00	18397
σ_*b*_1__	7.13	8.38	5.26	1.00	50000	6.90	7.28	0.92	1.00	18395
β_0_	3.70	0.17	3.70	1.00	1625	3.71	0.18	3.71	1.00	481
σ [1]	0.93	0.09	0.93	1.00	50000	0.93	0.09	0.93	1.00	30543
σ [2]	1.06	0.10	1.06	1.00	14474	1.06	0.10	1.05	1.00	50000
σ [3]	1.03	0.10	1.02	1.00	50000	1.04	0.10	1.03	1.00	50000
σ [4]	1.06	0.10	1.06	1.00	50000	1.06	0.10	1.05	1.00	50000
σ [5]	0.98	0.10	0.97	1.00	50000	0.97	0.09	0.97	1.00	50000
σ [6]	1.14	0.11	1.14	1.00	50000	1.14	0.11	1.14	1.00	50000

*β_0_ = the grand mean; **B_S_** = the matrix for sender effects; **B_R_** = the matrix for receiver effect; ρ_β_ = the correlation coefficient between sender effect and receiver effect; **B_D_** = the matrix for effect of distance metric; **Θ** the matrix for latent trait scores; ρ_θ_ = the correlation coefficient between two latent traits; σ = the matrix for variance of errors; ESS = effective sample size.*

Large receiver effects (0.65 under Model 1 and 0.74 under Model 2) were found for Item 6 (“Other work associates who must interact with this individual consider him/her to be trustworthy.”). Also, significant receiver effects (0.57 under Model 1 and 0.68 under Model 2) were found for Item 2 (“If I shared my problems with this person, I know (s)he would respond constructively and caringly.”). These two items showed strong abilities in differentiating both rating receivers’ and rating senders’ trait scores. In other words, in measuring interpersonal trust, students’ responses to these two items were highly sensitive indicators in capturing both sides’ trust. In contrast, the other four items responded strongly to the change of rating senders’ traits scores, but weakly to the score change of rating receivers, indicating more differentiative indicators for one member’s trait than for that of the other. Additionally, the effects of the distance metric produced under Model 2 were small (ranging from −0.09 to −0.02) for all six items, suggesting no items were influenced by the dissimilarity between students’ trust. Finally, a high correlation (0.81 under both models) between sender effects and receiver effects from the same trust trait was found, suggesting one’s general attitude toward interpersonal trust may play a highly similar role in their own ratings to others as in the ratings they received from others.

Overall, as with psychometric models, the LIDM can be used as a tool by which to score group members’ relational traits and to evaluate items’ characteristics in capturing different aspects of a dyadic interaction in measurement settings that feature mutual ratings within networks. These aspects include items’ abilities to differentiate rating-receivers’ and rating-senders’ traits for any given relational constructs, as well as the dissimilarities among dyad members’ traits. Researchers may need to decide for themselves which specific item characteristics ought to be preferred or valued in measuring a certain type of relationship. These decisions ought to be subject to the theories on the construct of interest as well as the purposes of measurement.

**FIGURE 3:**
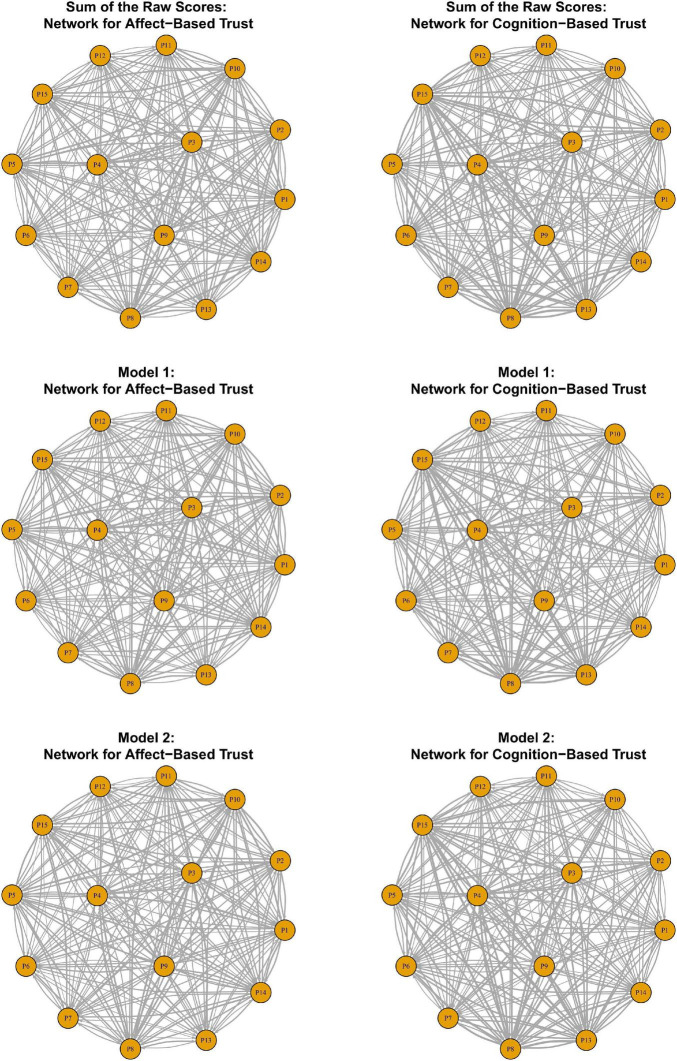
Affect-based trust networks and cognition-based trust networks generated from the raw scores and the estimates from Model 1 and Model 2.

Further, using the estimates of students’ trait scores, we calculated the predicted score for each observed response (excluding measurement error). The predicted scores represent students’ “true” responses to a given item at their latent trait level. A student’s predicted responses from the same subscale are summed up to quantify the “true” magnitude of the trait-level ties they have to others in the network, which can be interpreted using the original metric of the trust measure. We present these “true” magnitudes of each directed trait-level tie in Supplementary Table 1, which has been included in the Supplementary Materials. Figure 3 presents the affect-based trust networks and the cognition-based trust networks constructed by the sum of the raw response scores and the trait-level weighted ties under Model 1 and Model 2.

## Discussion

### On the Development of the Latent Interdependence Models

Psychometric relational data come from a variety of different research settings (e.g., “multiple” round-robin designs and block designs). The data we focused on in this paper are continuous psychometric data with a network structure (as illustrated by Table 1), which typically come from a setting in which each network member rates their relations with all others. As psychometric models, the goal of the LIDM is to score individuals’ relational traits and to evaluate the characteristics of the items. The estimates of model parameters can also be used to predict the magnitude of the connections among the group members along each latent dimension. These weighted connections construct univariate or multivariate social networks at a latent-trait level and can be interpreted using the metric of a given measure.

The LIDM adopt the idea of interdependence from the social relations model (SRM), in which a mutual-rating process is viewed as being influenced by both rating senders’ and receivers’ characteristics. However, they differ from SRMs in that they score everyone’s relational traits directly. Moreover, under the LIDM, the influence of ones’ traits on dyadic responses is conditional on an item’s characteristics—its ability to differentiate rating-senders’ and rating-receivers’ traits and the dissimilarities between traits. These item characteristics provide researchers with some useful perspectives, enabling them to evaluate how to measure a given relational construct properly. For instance, in measuring friendship, researchers may have an opportunity to evaluate the differences between using a “individual-focused” item (e.g., *“I like my friend.”*) and using a “dyad-focused” item (e.g., *“My friend and I like each other.”*).

In the LIDM, the basic units of modeling are dyads, which makes the LIDM distinct from other psychometric models (e.g., univariate or multivariate item response models) whose modeling units are single persons. For instance, in multivariate item response models, multiple latent traits capture an examinee’s set of abilities, with no need to involve the abilities of other individuals, nor any need to involve a component for the dissimilarity between two individuals’ abilities in the measurement or structural model.

Moreover, unlike traditional social network models (e.g., p* models and latent space model) that evolved from data-mining methodologies, the LIDM can be applied to theory-driven studies using psychometric relational data to represent a latent structure of relational constructs. By yielding accurate estimates for the latent traits and their covariance, the LIDM prepares social network researchers for further examinations of the mechanisms that underlie the formation of ties and for visualizing multivariate social networks at a latent-trait level.

To make the models estimable, constraints were placed onto certain parameters. In both models, the loadings conveying the effects of receiver’s latent traits were restricted to be positive, to have a fixed variance of one, to be equal across dyads, and to be item-specific. The variances of the random errors were also held equal across dyads and varied only across items. In addition, in Model 2, the effects of the distance metric were constrained to be negative and to be item-specific. By specifying these constraints, the number of parameters to be estimated does not rise as the network size grows. We note that although these proposed constraints and estimation procedures have been shown to be effective for continuous outcomes, further investigation is needed to generalize these constraints to other types of outcome data. It should be noted that with receiver effects being constrained to be positive, group members’ responses need to be scored in a proper way before entering analysis. That is, reverse scoring may be needed for some items to make sure a rating-sender’s higher score implies a rating-receiver’s higher trait score.

### On the Simulation Study

The objectives of the simulation study were to evaluate the parameterizations of the two proposed models and to investigate the effectiveness of model estimation at different network sizes. The results showed that, using the proposed Bayesian estimation procedures with uninformative priors, both models converged successfully for all simulated samples and that model parameters were accurately recovered under most conditions. These findings support the effectiveness of the parameterizations of both models. Moreover, when estimated with data that were generated from a different model, both models yielded relatively accurate results for the latent trait scores, suggesting a tolerance of both models to some model misspecification.

As expected, the accuracy of parameter recovery was improved when network size increased, suggesting that increasing network size can improve estimation accuracy. Since an increase in network size would burden group members by necessitating more ratings, researchers may face the trade-off between the quality of model estimation and the practicability of data collection. Additional non-round-robin data collection designs (where not all raters rate all others) need further research, as implications for missing data are not considered in this paper. Although producing small biases, the estimation from a small network with five or ten members was not satisfactorily accurate in general due to relatively large *RMSE* for certain parameters. We suggest that caution with respect to estimation accuracy when interpreting the results from the analyses with small networks. In addition, given a substantial improvement in the accuracy of parameter recovery when the network size grew from 10 to 20, a network size over 20 may suffice in terms of reaching satisfactory estimation accuracy under the parameter values studied here.

In the simulation study estimation procedures using Bayesian methods, weakly informative prior distributions were chosen for all parameters but the latent traits to maximally reflect the information from the data. It should be noted that in this study, the estimates of parameters are obtained *via* expected *a posteriori* (EAP) estimation. It has been noted that the EAP estimates and other Bayesian methods (e.g., MAP estimates) are inwardly biased with a tendency toward the mean of a given parameter in estimating traits under item response theory models (e.g., Wang, 2015; Feuerstahler, 2018). For future studies, a systematic evaluation of various estimation methods with LIDM is needed.

### Limitations and Future Studies

Despite evidence that supports the parametrization of the proposed models, we note some considerations on model development and the design of the simulation study. First, in this study, it was assumed that the dyadic relational response data were continuous. In fact, psychometric relational data can have multiple sample spaces. For instance, data collected using a behavior checklist may be binary. In such cases, generalized latent interdependence models with appropriate distributions and link functions could be used. The models can also be generalized to other types of data (e.g., ordinal data and count data). In developing the LIDM, we only considered the situations where each item only measures one relational trait and constrained the form of distance metric as the Euclidean metric. Future studies are needed to explore a more general form of the models that would apply for items measuring multiple traits and/or that include a more general distance metric (e.g., the Minkowski distance).

Second, the evidence from the simulation study does not guarantee the usefulness of the proposed models in other settings. In the simulation study, data were generated under a particular design with known dimensionality, in which eight items measured two latent traits (and each item measured only one latent trait). Although such a design seems to be practical for social and behavioral sciences research, sometimes researchers must compromise their design to address practical concerns. For instance, because group members could be burdened by a heavy item load, researchers may consider using only a few items to measure each relation in a network. Unfortunately, small numbers of items may cause estimation issues, such that extra constraints may need to be put onto the models. Further studies are needed to address these issues.

Finally, it should be noted that Model 1 is nested within Model 2 (given a zero effect of the distance metric). In practice, researchers can build their models starting from Model 1, with which not only could they score latent traits and evaluate item characteristics in terms of their sensitivity to trait change, but also understand which part of a dyad dominates the rating process. Moving to Model 2, researchers have an opportunity to test the effect of the interplay between dyad members’ latent characteristics. Empirically comparing the two models would involve a consideration of the information obtained from statistics (such as Bayes factors) or an examination of the highest density posterior credible intervals for the parameters of Model 2 that are not part of Model 1. Future studies are needed to derive and test Bayes factors for the LIDM.

## Concluding Remarks

Interpersonal relations can be studied at different levels. Researchers collect psychometric relational data within network settings to study those relational constructs that could be conceptualized as psychological processes with multiple latent dimensions. The development of the latent interdependence models (LIDM) is an effort to model psychometric data in social networks. The LIDM have the potential to be used in theory-driven studies to help explain the latent structure of any relational constructs. They produce estimates for group members’ relational traits and for the effects capturing items’ sensitivity to the change of traits and to the dissimilarity between a pair of members’ traits. Moreover, through deriving the magnitudes of the connections among group members, such an analytical strategy translates heavy-laden observed networks into practically analyzable multivariate networks at a latent-trait level. Further analyses (e.g., network properties calculation and multivariate networks visualization) could be done with the latent univariate or multivariate networks, opening the door to continued development.

## Data Availability Statement

The datasets presented in this study can be found in online repositories. The names of the repository/repositories and accession number(s) can be found below: DOI: 10.6084/m9.figshare.14925474.v4, reference number https://figshare.com/s/1c5bac0b88596795c3c4.

## Author Contributions

JT and LH contributed to the development of the proposed models, supervised the design of the study, and reviewed the manuscript. BH developed the models and estimation procedures, stimulated the data, performed the model evaluations, and wrote the manuscript. All authors contributed to the article and approved the submitted version.

## Conflict of Interest

The authors declare that the research was conducted in the absence of any commercial or financial relationships that could be construed as a potential conflict of interest.

## Publisher’s Note

All claims expressed in this article are solely those of the authors and do not necessarily represent those of their affiliated organizations, or those of the publisher, the editors and the reviewers. Any product that may be evaluated in this article, or claim that may be made by its manufacturer, is not guaranteed or endorsed by the publisher.

## Footnotes

1 Both mean-based estimates and median-based estimates were obtained. The results of parameter recovery were reported based on mean-based estimates. The results based on median-based estimates have been included in the Supplementary Tables M1, M2.
