# Supplementary material for: Modeling Psychometric Relational Data in Social Networks: Latent Interdependence Models
Source: Front Psychol. 2022 Apr 7;13:860837. doi: 10.3389/fpsyg.2022.860837 (PMC9021498; doi:10.3389/fpsyg.2022.860837)
Supplement: Supplementary file 1 [file Table_1.docx]

***Supplementary Material***

# Supplementary Tables

# Supplementary Tables

Table M1

Root-Mean-Squared Errors (*RMSE*s), Normalized Root-Mean-Squared Errors (*NRMSE*s), Biases and Coefficient of Determination ($R^{2}$) of Parameter Recovery (calculated using the median of posterior distributions; the Number of Simulations = 300)

| **Parameters** | **Network Size** | | | | | | | | | | | |
| --- | --- | --- | --- | --- | --- | --- | --- | --- | --- | --- | --- | --- |
|  | ***n = 5*** | | | | ***n = 10*** | | | | ***n* = 20** | | | |
|  | ***RMSE*** | ***NRMSE*** | ***Bias*** | $\boldsymbol{R}^{\boldsymbol{2}}$ | ***RMSE*** | ***NRMSE*** | ***Bias*** | $\boldsymbol{R}^{\boldsymbol{2}}$ | ***RMSE*** | ***NRMSE*** | ***Bias*** | $\boldsymbol{R}^{\boldsymbol{2}}$ |
| $\beta_{0}$ | .162  (.196) | .160  (.190) | .041  (.025) | .950  (.950) | .160  (.187) | .160  (.183) | .010  (.026) | .950  (.952) | .070  (.10) | .069  (.103) | .008  (.013) | .973  (.973) |
| $\boldsymbol{B}_{\mathbf{S}}$ | .192  (.264) | .179  (.267) | .063  (.030) | .951  (.949) | .220  (.250) | .216  (.250) | .045  (.080) | .957  (.957) | .134  (.130) | .129  (.131) | .042  (.050) | .967  (.970) |
| $\boldsymbol{B}_{\mathbf{R}}$ | .166  (.161) | .173  (.161) | .014  (.008) | .953  (.956) | .100  (.127) | .101  (.134) | .010  (.010) | .956  (.954) | .071  (.062) | .071  (.063) | .013  (.009) | .965  (.969) |
| $\rho_{\beta}$ | .209  (.230) | 3.483  (4.600) | .012  (.010) | .950  (.956) | .152  (.150) | 5.067  (7.500) | .016  (.010) | .952  (.952) | .100  (.093) | 2.500  (1.550) | .008  (.012) | .970  (.968) |
| $\boldsymbol{B}_{\boldsymbol{D}}$ | .230 | 2.30 | .026 | .950 | .209 | 1.900 | .030 | .954 | .060 | .545 | .003 | .975 |
| $\boldsymbol{\Theta}$ | .314  (.312) | .320  (.300) | −.020  (−.020) | .952  (.952) | .300  (.286) | .297  (.286) | −.016  (−.008) | .952  (.953) | .191  (.190) | .189  (.192) | −.007  (.006) | .965  (.967) |
| $\rho_{\theta}$ | .103  (.106) | 2.575  (2.650) | .024  (.024) | .948  (.950) | .116  (.094) | 5.800  (9.400) | .030  (.037) | .953  (.952) | .124  (.143) | 2.480  (4.767) | .062  (.060) | .965  (.967) |
| $\boldsymbol{\sigma}$ | .129  (.121) | .125  (.114) | −.024  (−.036) | .953  (.952) | .120  (.156) | .119  (.154) | −.041  (−.075) | .953  (.952) | .083  (.080) | .083  (.078) | −.010  (−.013) | .971  (.970) |
| *Notes*: * The number of simulations is 100 for a network size of 100. Results from Model 2 are presented in parentheses. | | | | | | | | | | | | |


Table M1

Root-Mean-Squared Errors (*RMSE*s), Normalized Root-Mean-Squared Errors (*NRMSE*s), Biases and Coefficient of Determination ($R^{2}$) of Parameter Recovery (calculated using the median of posterior distributions; the Number of Simulations = 300) (continued)

| **Parameters** | **Network Size** | | | | | | | |
| --- | --- | --- | --- | --- | --- | --- | --- | --- |
|  | ***n = 30*** | | | | ***n* = 100*** | | | |
|  | ***RMSE*** | ***NRMSE*** | ***Bias*** | $\boldsymbol{R}^{\boldsymbol{2}}$ | ***RMSE*** | ***NRMSE*** | ***Bias*** | $\boldsymbol{R}^{\boldsymbol{2}}$ |
| $\beta_{0}$ | .030  (.051) | .029  (.052) | −.009  (.009) | .982  (.987) | .009 (.007) | .009 (.007) | .009 (.006) | .996 (.996) |
| $\boldsymbol{B}_{\mathbf{S}}$ | .082  (.102) | .082  (.104) | .010  (.022) | .990  (.993) | .013 (.012) | .013 (.012) | .007 (.008) | .993 (.993) |
| $\boldsymbol{B}_{\mathbf{R}}$ | .030  (.043) | .031  (.044) | .007  (.006) | .991  (.991) | .009 (.010) | .009 (.010) | .009 (.008) | .993 (.994) |
| $\rho_{\beta}$ | .052  (.060) | .867  (3.000) | .007  (.006) | .988  (.987) | .010 (.010) | .500 (.333) | .004 (.003) | .992 (.994) |
| $\boldsymbol{B}_{\boldsymbol{D}}$ | .052 | .433 | .004 | .989 | .013 | .108 | .005 | .990 |
| $\boldsymbol{\Theta}$ | .093  (.121) | .093  (.121) | −.005  (.004) | .993  (.990) | .032 (.030) | .032 (.029) | .005 (.006) | .996 (.996) |
| $\rho_{\theta}$ | .080  (.123) | 1.333  (6.150) | −.092  (−.072) | .990  (.988) | .024 (.021) | .600 (1.050) | −.014 (.009) | .996 (.995) |
| $\boldsymbol{\sigma}$ | .040  (.052) | .040  (.053) | −.008  (−.010) | .990  (.994) | .007 (.007) | .007 (.007) | .000 (−.001) | .994 (.995) |

Table M2

Root-Mean-Squared Errors (*RMSE*s), Normalized Root-Mean-Squared Errors (*NRMSE*s), Biases, Coefficient of Determination ($R^{2}$) of Parameter Recovery from Cross-Estimation (calculated using the median of posterior distributions; the Number of Simulations = 300)

| **Parameters** | **Network Size** | | | | | | | | | | | |
| --- | --- | --- | --- | --- | --- | --- | --- | --- | --- | --- | --- | --- |
|  | ***n = 5*** | | | | ***n = 10*** | | | | ***n = 20*** | | | |
|  | *RMSE* | *NRMSE* | *Bias* | $R^{2}$ | *RMSE* | *NRMSE* | *Bias* | $R^{2}$ | *RMSE* | *NRMSE* | *Bias* | $R^{2}$ |
| $\boldsymbol{\Theta}$ | .372 (.320) | .380  (.308) | .024 (.022) | .946  (.950) | .360 (.332) | .356  (.332) | .013 (−.024) | .952  (.952) | .317 (.290) | .314  (.293) | .012 (.022) | .965  (.960) |
| $\rho_{\theta}$ | .220 (.156) | 5.500 (3.900) | .034 (.035) | .950  (.951) | .152 (.127) | 7.600 (12.700) | .030 (.034) | .953  (.950) | .146 (.142) | 2.920 (4.733) | −.007 (−.021) | .963  (.963) |
| *Notes*: * The number of simulations is 100 for a network size of 100. Results from Model 2 are presented in parentheses. $\boldsymbol{\Theta}$= the matrix for latent trait scores; $\rho_{\theta}$  *=* the correlation coefficient between two latent traits. | | | | | | | | | | | | |

Table M2

Root-Mean-Squared Errors (*RMSE*s), Normalized Root-Mean-Squared Errors (*NRMSE*s), Biases, Coefficient of Determination ($R^{2}$) of Parameter Recovery from Cross-Estimation (calculated using the median of posterior distributions; the Number of Simulations = 300) (continued)

| **Parameters** | **Network Size** | | | | | | | |
| --- | --- | --- | --- | --- | --- | --- | --- | --- |
|  | ***n = 30*** | | | | ***n* = 100*** | | | |
|  | ***RMSE*** | ***NRMSE*** | ***Bias*** | $\boldsymbol{R}^{\boldsymbol{2}}$ | ***RMSE*** | ***NRMSE*** | ***Bias*** | $\boldsymbol{R}^{\boldsymbol{2}}$ |
| $\boldsymbol{\Theta}$ | .210 (.152) | .210 (.152) | −.005 (−.005) | .990  (.988) | .010 (.010) | .010 (.010) | .006 (.007) | .994 (.993) |
| $\rho_{\theta}$ | .109 (.097) | 1.817 (4.850) | −.014 (.009) | .989  (.991) | .034 (.036) | .850 (1.800) | −.015 (.009) | .994 (.996) |

Table S1

Magnitudes of Connections among Actors along Two Interpersonal Trust Dimensions

| Dyads | **Model 1** | | | |  | **Model 2** | | | |
| --- | --- | --- | --- | --- | --- | --- | --- | --- | --- |
|  | **Affect-Based** | | **Cognition-Based** | |  | **Affect-Based** | | **Cognition-Based** | |
| (A,B) | **A to B** | **B to A** | **A to B** | **B to A** |  | **A to B** | **B to A** | **A to B** | **B to A** |
| (1,2) | 11.58 | 12.60 | 9.54 | 10.52 |  | 12.15 | 13.11 | 10.65 | 7.65 |
| (1,3) | 11.41 | 11.53 | 8.86 | 9.02 |  | 12.32 | 11.34 | 7.61 | 8.68 |
| (1,4) | 10.86 | 7.99 | 9.88 | 11.28 |  | 9.40 | 7.78 | 9.46 | 10.74 |
| (1,5) | 11.59 | 12.63 | 9.20 | 9.77 |  | 11.22 | 12.91 | 11.74 | 7.88 |
| (1,6) | 10.92 | 8.36 | 9.58 | 10.62 |  | 11.37 | 6.43 | 8.95 | 9.90 |
| (1,7) | 11.02 | 9.00 | 8.58 | 8.40 |  | 9.53 | 9.79 | 6.34 | 10.20 |
| (1,8) | 11.90 | 14.65 | 12.60 | 17.29 |  | 8.31 | 13.89 | 9.97 | 16.93 |
| (1,9) | 11.26 | 10.52 | 7.81 | 6.70 |  | 11.65 | 9.23 | 8.12 | 8.74 |
| (1,10) | 11.87 | 14.46 | 9.51 | 10.46 |  | 9.82 | 17.28 | 9.96 | 9.01 |
| (1,11) | 11.30 | 10.80 | 8.80 | 8.89 |  | 11.77 | 9.47 | 6.98 | 8.63 |
| (1,12) | 10.82 | 7.71 | 7.97 | 7.06 |  | 11.86 | 8.15 | 7.22 | 8.92 |
| (1,13) | 10.91 | 8.30 | 11.51 | 14.87 |  | 11.80 | 9.48 | 14.48 | 14.17 |
| (1,14) | 11.69 | 13.28 | 9.02 | 9.38 |  | 13.36 | 15.78 | 12.80 | 9.32 |
| (1,15) | 11.54 | 12.35 | 12.03 | 16.02 |  | 12.03 | 9.94 | 12.65 | 16.63 |
| (2,3) | 12.62 | 11.72 | 10.66 | 9.83 |  | 11.79 | 11.84 | 13.43 | 4.22 |
| (2,4) | 12.07 | 8.18 | 11.68 | 12.09 |  | 12.33 | 10.86 | 12.08 | 15.46 |
| (2,5) | 12.79 | 12.82 | 11.00 | 10.59 |  | 9.80 | 9.44 | 13.31 | 9.89 |
| (2,6) | 12.13 | 8.55 | 11.38 | 11.44 |  | 14.01 | 8.45 | 7.70 | 9.16 |
| (2,7) | 12.23 | 9.19 | 10.38 | 9.21 |  | 11.64 | 10.03 | 6.72 | 10.43 |
| (2,8) | 13.11 | 14.84 | 14.40 | 18.11 |  | 12.85 | 13.26 | 15.76 | 19.59 |
| (2,9) | 12.46 | 10.71 | 9.61 | 7.51 |  | 11.17 | 10.54 | 9.72 | 10.38 |
| (2,10) | 13.08 | 14.64 | 11.31 | 11.27 |  | 14.15 | 16.14 | 10.38 | 9.81 |
| (2,11) | 12.51 | 10.99 | 10.60 | 9.70 |  | 11.35 | 9.71 | 10.60 | 8.41 |
| (2,12) | 12.02 | 7.90 | 9.77 | 7.87 |  | 12.80 | 4.50 | 9.79 | 7.91 |
| (2,13) | 12.12 | 8.49 | 13.31 | 15.69 |  | 10.16 | 7.12 | 15.30 | 14.85 |
| (2,14) | 12.90 | 13.46 | 10.82 | 10.19 |  | 11.11 | 12.84 | 11.23 | 9.55 |
| (2,15) | 12.75 | 12.54 | 13.82 | 16.83 |  | 13.33 | 10.12 | 11.57 | 15.02 |
| (3,4) | 11.00 | 8.01 | 10.17 | 11.41 |  | 10.00 | 8.10 | 8.74 | 9.61 |
| (3,5) | 11.73 | 12.65 | 9.49 | 9.90 |  | 11.59 | 10.88 | 10.31 | 9.93 |
| (3,6) | 11.06 | 8.38 | 9.88 | 10.75 |  | 10.30 | 10.26 | 13.24 | 11.61 |
| (3,7) | 11.16 | 9.03 | 8.87 | 8.53 |  | 12.57 | 8.60 | 10.37 | 9.32 |
| (3,8) | 12.04 | 14.67 | 12.90 | 17.43 |  | 14.05 | 14.77 | 9.28 | 17.99 |
| (3,9) | 11.40 | 10.54 | 8.10 | 6.83 |  | 8.27 | 10.19 | 6.95 | 7.86 |
| (3,10) | 12.01 | 14.48 | 9.80 | 10.59 |  | 11.63 | 16.85 | 9.79 | 9.30 |
| (3,11) | 11.44 | 10.82 | 9.09 | 9.02 |  | 8.77 | 11.81 | 11.00 | 7.00 |
| (3,12) | 10.96 | 7.73 | 8.26 | 7.19 |  | 7.63 | 7.51 | 4.90 | 6.63 |
| (3,13) | 11.05 | 8.32 | 11.80 | 15.01 |  | 16.45 | 8.72 | 12.36 | 15.86 |
| (3,14) | 11.83 | 13.30 | 9.32 | 9.51 |  | 12.76 | 12.73 | 10.64 | 6.22 |
| (3,15) | 11.68 | 12.37 | 12.32 | 16.15 |  | 13.20 | 14.02 | 14.91 | 12.29 |
| (4,5) | 8.19 | 12.10 | 11.75 | 10.93 |  | 6.05 | 10.57 | 10.12 | 12.73 |
| (4,6) | 7.52 | 7.83 | 12.13 | 11.78 |  | 7.58 | 2.30 | 9.39 | 14.37 |
| (4,7) | 7.62 | 8.47 | 11.13 | 9.55 |  | 10.22 | 9.90 | 12.94 | 8.84 |
| (4,8) | 8.50 | 14.12 | 15.15 | 18.45 |  | 9.51 | 14.74 | 16.46 | 17.13 |
| (4,9) | 7.86 | 9.99 | 10.36 | 7.85 |  | 5.27 | 10.89 | 7.81 | 7.57 |
| (4,10) | 8.47 | 13.92 | 12.06 | 11.61 |  | 6.89 | 15.58 | 10.95 | 9.57 |
| (4,11) | 7.90 | 10.27 | 11.35 | 10.04 |  | 11.35 | 8.03 | 8.86 | 9.51 |
| (4,12) | 7.42 | 7.18 | 10.52 | 8.21 |  | 8.61 | 8.23 | 7.28 | 9.54 |
| (4,13) | 7.51 | 7.77 | 14.06 | 16.03 |  | 6.80 | 11.56 | 15.71 | 15.32 |
| (4,14) | 8.29 | 12.74 | 11.57 | 10.53 |  | 6.97 | 6.64 | 11.62 | 10.82 |
| (4,15) | 8.14 | 11.82 | 14.58 | 17.17 |  | 8.04 | 13.64 | 16.18 | 16.06 |
| (5,6) | 12.15 | 8.55 | 10.63 | 11.10 |  | 13.72 | 10.13 | 10.40 | 11.70 |
| (5,7) | 12.25 | 9.20 | 9.62 | 8.87 |  | 13.26 | 8.64 | 9.66 | 11.62 |
| (5,8) | 13.14 | 14.85 | 13.65 | 17.77 |  | 12.72 | 14.45 | 14.11 | 16.35 |
| (5,9) | 12.49 | 10.72 | 8.85 | 7.17 |  | 10.79 | 11.39 | 7.98 | 7.61 |
| (5,10) | 13.11 | 14.65 | 10.56 | 10.93 |  | 13.58 | 17.63 | 11.64 | 9.79 |
| (5,11) | 12.54 | 11.00 | 9.85 | 9.36 |  | 14.13 | 12.57 | 12.49 | 13.65 |
| (5,12) | 12.05 | 7.91 | 9.02 | 7.53 |  | 12.00 | 7.22 | 9.12 | 8.83 |
| (5,13) | 12.14 | 8.50 | 12.55 | 15.35 |  | 12.43 | 11.08 | 16.24 | 16.05 |
| (5,14) | 12.92 | 13.47 | 10.07 | 9.85 |  | 14.63 | 13.06 | 9.38 | 10.47 |
| (5,15) | 12.78 | 12.54 | 13.07 | 16.49 |  | 15.04 | 11.82 | 13.73 | 17.68 |
| (6,7) | 7.98 | 8.53 | 10.47 | 9.26 |  | 9.86 | 10.63 | 12.23 | 7.49 |
| (6,8) | 8.87 | 14.18 | 14.50 | 18.15 |  | 8.19 | 15.36 | 14.55 | 19.59 |
| (6,9) | 8.22 | 10.05 | 9.70 | 7.56 |  | 7.85 | 13.10 | 9.77 | 9.04 |
| (6,10) | 8.84 | 13.98 | 11.41 | 11.32 |  | 7.56 | 12.81 | 12.22 | 12.33 |
| (6,11) | 8.27 | 10.33 | 10.70 | 9.75 |  | 5.34 | 13.16 | 9.73 | 12.38 |
| (6,12) | 7.78 | 7.24 | 9.87 | 7.92 |  | 7.47 | 8.21 | 7.48 | 7.30 |
| (6,13) | 7.87 | 7.83 | 13.40 | 15.73 |  | 7.02 | 6.58 | 12.42 | 15.49 |
| (6,14) | 8.65 | 12.80 | 10.92 | 10.24 |  | 9.28 | 11.61 | 9.56 | 13.04 |
| (6,15) | 8.51 | 11.87 | 13.92 | 16.88 |  | 4.24 | 10.69 | 14.98 | 15.35 |
| (7,8) | 9.51 | 14.28 | 12.28 | 17.14 |  | 9.89 | 9.98 | 10.00 | 18.10 |
| (7,9) | 8.87 | 10.15 | 7.48 | 6.55 |  | 8.68 | 11.25 | 7.26 | 6.66 |
| (7,10) | 9.48 | 14.08 | 9.18 | 10.31 |  | 7.87 | 11.13 | 8.85 | 14.79 |
| (7,11) | 8.91 | 10.43 | 8.47 | 8.74 |  | 9.44 | 10.31 | 10.72 | 5.48 |
| (7,12) | 8.43 | 7.34 | 7.64 | 6.91 |  | 5.99 | 5.07 | 7.94 | 7.11 |
| (7,13) | 8.52 | 7.93 | 11.18 | 14.72 |  | 6.13 | 5.02 | 13.27 | 14.33 |
| (7,14) | 9.30 | 12.90 | 8.69 | 9.23 |  | 10.14 | 12.63 | 7.56 | 9.53 |
| (7,15) | 9.15 | 11.97 | 11.70 | 15.87 |  | 5.01 | 15.12 | 7.87 | 12.44 |
| (8,9) | 14.52 | 11.03 | 16.37 | 10.58 |  | 16.65 | 9.22 | 19.41 | 10.03 |
| (8,10) | 15.13 | 14.97 | 18.08 | 14.34 |  | 15.56 | 15.96 | 18.33 | 12.54 |
| (8,11) | 14.56 | 11.31 | 17.37 | 12.77 |  | 15.38 | 11.85 | 18.01 | 15.33 |
| (8,12) | 14.08 | 8.22 | 16.54 | 10.93 |  | 15.98 | 10.26 | 16.26 | 10.67 |
| (8,13) | 14.17 | 8.81 | 20.07 | 18.75 |  | 13.07 | 8.26 | 20.61 | 15.18 |
| (8,14) | 14.95 | 13.79 | 17.59 | 13.26 |  | 15.81 | 12.66 | 18.21 | 11.65 |
| (8,15) | 14.80 | 12.86 | 20.59 | 19.89 |  | 13.28 | 12.76 | 19.69 | 20.20 |
| (9,10) | 11.00 | 14.32 | 7.48 | 9.54 |  | 11.49 | 13.81 | 5.52 | 7.07 |
| (9,11) | 10.43 | 10.67 | 6.77 | 7.97 |  | 7.70 | 13.71 | 6.30 | 9.42 |
| (9,12) | 9.95 | 7.58 | 5.94 | 6.14 |  | 10.40 | 6.56 | 4.04 | 5.43 |
| (9,13) | 10.04 | 8.17 | 9.48 | 13.96 |  | 11.84 | 9.55 | 10.61 | 15.15 |
| (9,14) | 10.82 | 13.14 | 6.99 | 8.46 |  | 13.13 | 11.23 | 6.02 | 10.14 |
| (9,15) | 10.67 | 12.21 | 10.00 | 15.10 |  | 8.41 | 12.61 | 11.39 | 13.85 |
| (10,11) | 14.36 | 11.28 | 10.53 | 9.67 |  | 14.86 | 11.01 | 10.35 | 7.60 |
| (10,12) | 13.88 | 8.19 | 9.70 | 7.84 |  | 15.56 | 6.54 | 6.78 | 7.08 |
| (10,13) | 13.97 | 8.78 | 13.24 | 15.66 |  | 12.36 | 10.48 | 14.10 | 13.63 |
| (10,14) | 14.75 | 13.75 | 10.75 | 10.16 |  | 16.13 | 12.29 | 8.92 | 9.40 |
| (10,15) | 14.61 | 12.83 | 13.76 | 16.80 |  | 14.96 | 12.45 | 14.29 | 19.71 |
| (11,12) | 10.23 | 7.62 | 8.13 | 7.13 |  | 6.50 | 7.99 | 8.31 | 8.19 |
| (11,13) | 10.32 | 8.21 | 11.67 | 14.95 |  | 11.26 | 9.79 | 14.42 | 14.31 |
| (11,14) | 11.10 | 13.18 | 9.18 | 9.45 |  | 11.47 | 15.02 | 9.68 | 13.21 |
| (11,15) | 10.95 | 12.26 | 12.19 | 16.09 |  | 11.46 | 11.94 | 13.68 | 17.75 |
| (12,13) | 7.23 | 7.73 | 9.84 | 14.12 |  | 8.82 | 7.91 | 11.44 | 14.44 |
| (12,14) | 8.01 | 12.70 | 7.35 | 8.62 |  | 5.23 | 11.21 | 3.70 | 10.42 |
| (12,15) | 7.86 | 11.77 | 10.36 | 15.26 |  | 8.92 | 12.80 | 9.32 | 13.49 |
| (13,14) | 8.60 | 12.79 | 15.17 | 12.16 |  | 7.96 | 12.27 | 15.40 | 13.08 |
| (13,15) | 8.45 | 11.86 | 18.17 | 18.80 |  | 7.71 | 11.01 | 18.22 | 18.69 |
| (14,15) | 13.42 | 12.64 | 12.68 | 16.31 |  | 13.27 | 10.36 | 10.67 | 14.14 |

# JAGS Syntax

The JAGS syntax we used to fit the two proposed models on the simulated data are shown below. We define Q as a matrix to describe the item–trait relations. For the situations we considered in the simulation study, Q is an 8 $\times$ 2 matrix with 0 and 1 as its elements, indicating which trait an item is designed to measure. We define d1 and d2 as two vectors containing the indices for all rating-senders and all rating-receivers, respectively, from a total of $\binom{n}{2}$ dyads. For instance, if [2,3] is the *i*^th^ dyad out of a group of *n* actors, then the *i*^th^ element of d1 will be 2 and the *i*^th^ element of d2 will be 3.

### Model 1

model{

#######################

## Measurement Model ###

#######################

for (i in 1:ndyad){

for (j in 1:nitem){

y1[i,j] ~ dnorm(mu1[i,j],tau.y[j])

y2[i,j] ~ dnorm(mu2[i,j],tau.y[j])

}

}

for (i in 1:ndyad){

for (j in 1:nitem){

mu1[i,j] <- alpha+beta1[j]*theta[d1[i],1]*Q[j,1]+beta1[j]*theta[d1[i],2]*Q[j,2]+

beta2[j]*theta[d2[i],1]*Q[j,1]+beta2[j]*theta[d2[i],2]*Q[j,2]

mu2[i,j] <- alpha+beta1[j]*theta[d2[i],1]*Q[j,1]+beta1[j]*theta[d2[i],2]*Q[j,2]+

beta2[j]*theta[d1[i],1]*Q[j,1]+beta2[j]*theta[d1[i],2]*Q[j,2]

}

}

#######################

### Structural Model #####

#######################

for (i in 1:nnode){theta[i,1:2] ~ dmnorm(mu.theta,tau.theta[,])}

for (i in 1:2){mu.theta[i] <- 0}

for (i in 1:2){L.theta[i,i] <- 1}

Rho ~ dunif(0,1)

L.theta[2,1] <- Rho

L.theta[1,2] <- 0

sigma.theta <- L.theta %*% t(L.theta)

tau.theta <- inverse(sigma.theta)

#############################

####priors on all effects #########

#############################

beta0 ~ dnorm(0,0.1)

for (i in 1:nitem){

tau.y[i] ~ dgamma(9,4)

sigma.y[i] <- 1/tau.y[i]

beta2[i] ~ dnorm(mu.beta2,tau.beta2)T(0,)

beta1[i] ~ dnorm(mu.beta1+lam*(beta2[i]-mu.beta2),tau.beta1)

}

mu.beta1 <- 0

mu.beta2 <- 0

lam ~ dnorm(0,0.0001)

var2 <- 1/tau.beta2

var1 <- 1/tau.beta1 + (lam^2)*var2

tau.beta2 <- pow(sigma.beta2,-2)

tau.beta1 <- pow(sigma.beta1,-2)

sigma.beta2 <- 1

sigma.beta1 ~ dunif(0,1000)

Rho_b <- lam*sqrt(var2/var1)

}

###Model 2

model{

#######################

## Measurement Model ###

#######################

for (i in 1:ndyad){

for (j in 1:nitem){

y1[i,j] ~ dnorm(mu1[i,j],tau.y[j])

y2[i,j] ~ dnorm(mu2[i,j],tau.y[j])

}

}

for (i in 1:ndyad){

for (j in 1:nitem){

mu1[i,j] <- alpha+beta1[j]*theta[d1[i],1]*Q[j,1]+beta1[j]*theta[d1[i],2]*Q[j,2]+

beta2[j]*theta[d2[i],1]*Q[j,1]+beta2[j]*theta[d2[i],2]*Q[j,2]-

beta3[j]*pow(pow(theta[d1[i],1]-theta[d2[i],1],2)+ pow(theta[d1[i],2]-theta[d2[i],2],2),1/2)

mu2[i,j] <- alpha+beta1[j]*theta[d2[i],1]*Q[j,1]+beta1[j]*theta[d2[i],2]*Q[j,2]+

beta2[j]*theta[d1[i],1]*Q[j,1]+beta2[j]*theta[d1[i],2]*Q[j,2]-

beta3[j]*pow(pow(theta[d2[i],1]-theta[d1[i],1],2)+ pow(theta[d2[i],2]-theta[d1[i],2],2),1/2)

}

}

#######################

### Structural Model #####

#######################

for (i in 1:nnode){theta[i,1:2] ~ dmnorm(mu.theta,tau.theta[,])}

for (i in 1:2){mu.theta[i] <- 0}

for (i in 1:2){L.theta[i,i] <- 1}

Rho ~ dunif(0,1)

L.theta[2,1] <- Rho

L.theta[1,2] <- 0

sigma.theta <- L.theta %*% t(L.theta)

tau.theta <- inverse(sigma.theta)

#############################

### priors on all effects #########

#############################

beta0 ~ dnorm(0,0.1)

for (i in 1:nitem){

tau.y[i] ~ dgamma(9,4)

sigma.y[i] <- 1/tau.y[i]

beta3[i] ~ dlnorm(0,0.1)

beta2[i] ~ dnorm(mu.beta2,tau.beta2)T(0,)

beta1[i] ~ dnorm(mu.beta1+lam*(beta2[i]-mu.beta2),tau.beta1)

}

mu.beta1 <- 0

mu.beta2 <- 0

lam ~ dnorm(0,0.0001)

var2 <- 1/tau.beta2

var1 <- 1/tau.beta1 + (lam^2)*var2

tau.beta2 <- pow(sigma.beta2,-2)

tau.beta1 <- pow(sigma.beta1,-2)

sigma.beta2 <- 1

sigma.beta1 ~ dunif(0,1000)

Rho_b <- lam*sqrt(var2/var1)

}
